# miR-708-5p enhances erlotinib/paclitaxel efficacy and overcomes chemoresistance in lung cancer cells

**DOI:** 10.18632/oncotarget.27840

**Published:** 2020-12-22

**Authors:** Nicholas J. Monteleone, Carol S. Lutz

**Affiliations:** ^1^Department of Microbiology, Biochemistry, and Molecular Genetics, Rutgers Biomedical & Health Sciences, New Jersey Medical School, School of Graduate Studies, Newark, NJ 07103, USA

**Keywords:** miR-708-5p, miR-708, erlotinib, paclitaxel, chemoresistance

## Abstract

Lung cancer is a collection of aggressive tumors generally not diagnosed until late-stage, resulting in high mortality rates. The vast majority of non-small cell lung cancer (NSCLC) patients undergo combinatory chemotherapeutic treatment, which initially reduces tumor growth, but frequently becomes ineffective due to toxicity and resistance. Researchers have identified multiple signaling pathways involved in lung cancer chemoresistance, including cyclooxygenase-2 (COX-2)/microsomal prostaglandin E synthase-1 (mPGES-1) derived prostaglandin E2 (PGE_2_). While COX-2 inhibitors have shown promise in the clinic, their use is limited due to severe side effects. One novel approach to effectively suppress COX-2 signaling is through microRNA (miRNA). MiRNAs are small-noncoding RNAs commonly misexpressed in cancer. One tumor suppressive miRNA, miR-708-5p, has been shown to repress pro-resistant signaling pathways, including COX-2 and mPGES-1. Here, we demonstrate that chemotherapies reduce COX-2 expression, possibly through induction of miR-708-5p. Moreover, combination treatment of erlotinib (ERL) or paclitaxel (PAC) with miR-708-5p enhances COX-2 and mPGES-1 protein suppression. We also show that combination chemotherapeutic and miR-708-5p treatment intensifies the anti-proliferative and pro-apoptotic effects of ERL and PAC. We also created ERL and PAC resistant lung cancer cell lines, which have increased COX-2 expression and diminished miR-708-5p levels compared to naïve lung cancer cells. While ERL and PAC treatments do not alter resistant cell phenotype alone, combination treatment with miR-708-5p partially restores the chemotherapies’ anti-proliferative effects and fully restores their pro-apoptotic qualities. These data suggest miR-708-5p may have potential combinatory therapeutic value to more efficaciously treat lung tumors while overcoming chemoresistance.

## INTRODUCTION

The Arachidonic Acid (AA) metabolic pathway is a lipid signaling pathway involved in homeostasis, development, and immune regulation [1]. The AA pathway has been implicated in numerous diseases, including autoimmunity, neurodegeneration, and cancer [1]. AA is an omega-6 20-carbon poly-unsaturated fatty acid found within the cytoplasm and membranes of the cell [2]. Free cytosolic AA is metabolized by one of two Cyclooxygenases (COXs): Cyclooxygenase-1 (COX-1) or Cyclooxygenase-2 (COX-2), which both convert AA to Prostaglandin H2 (PGH_2_), a short-lived intermediary product. COX-2 is inducible and is associated with disease while COX-1 is important for homeostatic prostaglandin production [3]. While there are three Prostaglandin E synthases (PGES) that convert PGH_2_ to PGE_2_ in mammals, microsomal PGES-1 (mPGES-1) is functionally coupled and co-expressed with COX-2 [4–9]. Once extracellular, PGE_2_ acts in an autocrine and paracrine fashion to regulate hematopoietic stem cell regeneration, inflammation, and gut integrity [10, 11]. While the COX-2/mPGES-1/PGE_2_ pathway has important homeostatic and immune-related functions, dysregulation of this signaling axis has been shown to have profound roles in cancer.

COX-2 is often constitutively expressed in many tumors and is associated with decreased survival rates. Long-term use of non-selective COX inhibitors, such as aspirin, decreases cancer rates [12–14]. mPGES-1 has also been shown to be overexpressed in cancer, and knockdown of mPGES-1 prevented tumor growth in breast and lung cancer *in vivo* [15–18]. Enhanced production of COX-2/mPGES-1-derived PGE_2_ promotes proliferation, invasion, survival, angiogenesis, and immune evasion in cancer [19]. PGE_2_ exerts its pro-tumorigenic functions mainly through stimulation of mitogen activated protein kinase (MAPK), Phosphoinositide 3-kinase (PI3K), nuclear factor kappa-light-chain-enhancer of activated B cells (NF-kB), and β-catenin signaling pathways [20–40]. Recently, researchers have also discovered that COX-2/mPGES-1-derived PGE_2_ also regulates cancer stem cell (CSC) renewal and chemoresistance [41].

COX-2 has been shown to be upregulated in chemotherapeutic resistant ovarian, lung, and colorectal cancer cells [42–44]. While some cancer treatments may induce COX-2, many cancer cells already express high levels of COX-2 prior to therapy, indicating that AA signaling promotes intrinsic resistance as well. COX-2 positively regulates expression of the efflux pump Multidrug Resistant Protein 4 (MRP4), which pumps PGE_2_ as well small molecule chemotherapies into the extracellular space [43, 44]. Studies have also identified the COX-2/mPGES-1/PGE_2_ signaling axis to be important in maintaining CSC populations, primarily by activating WNT signaling [41, 44–47]. Several studies and clinical trials using combination therapies of erlotinib, gemcitabine, paclitaxel, or platinum-based therapies have also shown synergistic effects with COX-2 inhibitors [46, 48–50]. It is important to note that high COX-2/PGE_2_ levels at baseline was a prognostic marker for therapeutic response in these studies. Hence it appears that tumors already expressing COX-2 are more likely to respond to combinatory chemotherapy plus COX-2 inhibitors than non-COX-2 expressing tumors. While small-molecule inhibitors have shown promise in the clinic, it is crucial to develop novel therapeutics that more fully target the pro-tumorigenic phenotype.

One way to regulate the AA pathway is through microRNA (miRNA). miRNAs are a class of conserved small non-coding RNAs that regulate gene expression post-transcriptionally [51, 52]. miRNAs are involved in a host of biological processes, from growth and development to homeostasis and the immune response [53, 54]. miRNAs are commonly dysregulated in cancer and can act as tumor suppressors or oncomiRs. We recently showed that one miRNA, miR-708-5p (miR-708), targets both the *Cox-2* and *mPGES-1* 3’ untranslated regions (UTRs) in lung cancer cells, resulting in decreased PGE_2_ levels [55]. Moreover, we demonstrated that miR-708 suppressed proliferation, survival, and migration of lung cancer cells, which could partially be contributed to its targeting of *Cox-2* and *mPGES-1*. Previously studies have shown that miR-708 also suppresses expression of various CSC markers, including CD34, CD44, CD117, Oct4, Aldehyde Dehydrogenase 1 Family Member A2 (ALDH1A2), and NANOG [56, 57]. Furthermore, several investigations concluded that miR-708 has multiple functions in reducing and overcoming chemoresistance [58–60]. Given these data, we aimed to study the role of AA signaling and its suppression by miR-708 in lung cancer cell chemoresistance.

Here, we demonstrate that chemotherapies induce miR-708-5p expression, which may be through p53 and CHOP. We also show that chemotherapies reduce COX-2 expression. Moreover, combination treatment of ERL or PAC with miR-708-5p enhanced the reduction in COX-2 and mPGES-1 protein expression. We also concluded that combination chemotherapeutic and miR-708-5p treatment intensified the anti-proliferative and pro-apoptotic effects greater than each therapy alone. Next, we created ERL and PAC resistant lung cancer cell lines. Chemoresistant cells had increased baseline COX-2 protein expression as well as reduced miR-708-5p expression compared to naïve lung cancer cells. While ERL and PAC treatments did not alter cell phenotype alone, combination treatment with miR-708-5p partially restored the chemotherapies’ anti-proliferative effects and fully restored their pro-apoptotic qualities.

## RESULTS

### miR-708-5p, COX-2, and mPGES-1 expression are regulated by ERL, PAC and DEX in lung cancer cells

**Figure 1:**
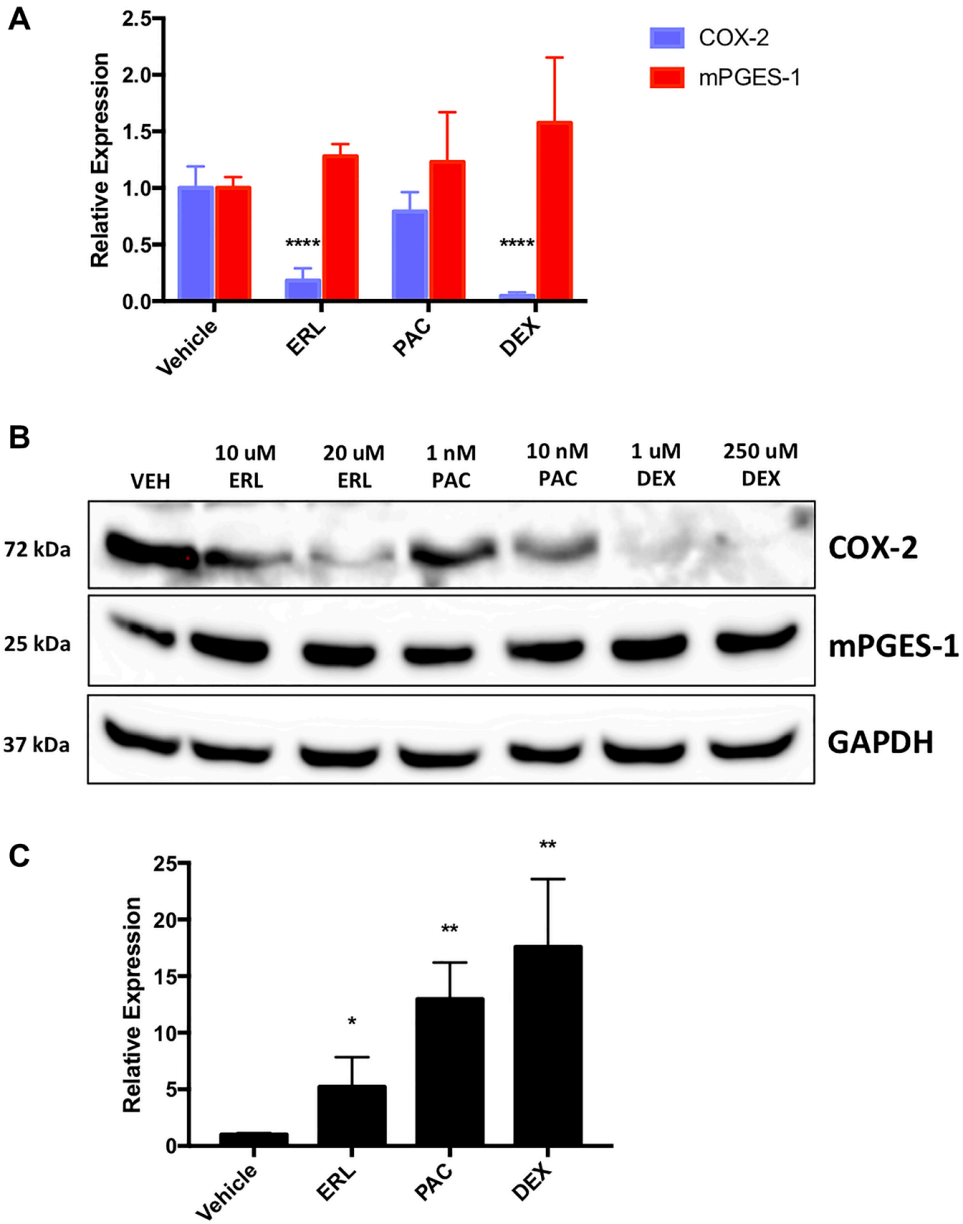
Chemotherapies regulate naïve lung cancer cell COX-2, mPGES-1 and miR-708-5p expression. (**A**) RT-qPCR of COX-2 (blue) and mPGES-1 (red) mRNA expression from A549 cells treated with vehicle, 20 uM ERL, 10 nM PAC, or 250 uM DEX for 24 hours. (**B**) Representative western blot of COX-2 and mPGES-1 protein expression in A549 cells treated with vehicle, 10/20 uM ERL, 1/10 nM PAC, or 1/250 uM DEX for 24 hours. GAPDH served as a loading control. (**C**) RT-qPCR of A549 cells treated with vehicle, 20 uM ERL, 10 nM PAC, or 250 uM DEX for 24 hours. COX-2 and mPGES-1 were normalized to GAPDH mRNA expression while miR-708-5p expression was normalized to mature miR-15a and analyzed using the 2- ΔΔCT method. ^*^ *p* < .05, ^**^ *p* < 0.01, ^****^ *p* < .0001, *n* ≥ 3.

Researchers have previously described multiple functions for miR-708-5p in chemoresistance. COX-2 and PGE_2_ also have well documented roles in promoting resistance in cancer. Additionally, we have shown that COX-2 and miR-708-5p expression is inversely correlated in lung cancer cells and tumors [55]. Therefore, we examined various chemoresistant aspects of miR-708-5p and its regulation of COX-2/mPGES-1 derived PGE_2_ in lung cancer cells. We tested the ability of ERL, PAC, and Dexamethasone (DEX) to modulate miR-708-5p and AA signaling in lung cancer cells. We treated A549 cells with ERL, PAC, or DEX for 48 hours and measured changes in mature COX-2 and mPGES-1 expression. We found that COX-2 mRNA was significantly decreased after ERL and DEX treatment compared to a vehicle control, whereas mPGES-1 mRNA was unchanged after all treatments (Figure 1A). We also measured COX-2 and mPGES-1 protein expression and observed similar results to RT-qPCR data (Figure 1B). Next, we investigated changes in miR-708-5p expression after treatment with ERL, PAC, and DEX. We observed significantly higher miR-708-5p expression in A549 cells 24 hours after treatment of each therapy (Figure 1C, *p* < 0.05, *n* = 3). Given these results, we explored transcription factors that may be regulating chemotherapeutic-induced miR-708-5p expression.

We examined known regulators of miR-708-5p and their correlation to miR-708-5p expression in NSCLC, LUAD, and LUSC patients. In the broader NSCLC subtype, every known regulator was significantly correlated with miR-708-5p expression (Table 1). Within NSCLC, the mRNA expression of transcription factors in LUAD were not significantly correlated with miR-708-5p expression, but transcription factor mRNA expression in LUSC tumors were highly correlated with miR-708-5p expression (Table 1). More specifically, CHOP was significantly positively correlated with miR-708-5p expression in NSCLC (Table 1, *p* = 0.207, *R*^2^ = 0.0418, *p* = 2.18 × 10^-11^) and LUSC (Table 1, *p* = 0.188, *R*^2^ = 0.0333, *p* = 2.32 × 10^-5^) tumors. It was previously shown that CHOP also induces p53, the most commonly mutated tumor suppressor in cancer [61]. Given the importance of p53 in tumorigenesis, we decided to investigate its relationship with miR-708-5p. We discovered that p53 mRNA expression was positively correlated with miR-708-5p expression in NSCLC, LUAD, and LUSC tumors (Table 2). Given CHOP’s previously defined regulation of miR-708-5p, as well as CHOP and p53’s profound roles in apoptosis, we examined if ERL, PAC, and DEX were regulating CHOP and p53 expression in lung cancer cells.

**Table 1:** miR-708-5p expression correlates with known miR-708-p regulators in lung cancer tumors

Gene	miRNA	NSCLC		LUAD		LUSC	
		Correlation	*p* value	Correlation	*p* value	Correlation	*p* value
CHOP^^^	miR-708	0.207	2.181E-11	0.0445	0.3085	0.188	0.00002323
*GRα*^^^	miR-708	*–0.236*	*1.825E-14*	–0.0579	0.1851	–*0.203*	*0.000004526*
MYC^^^	miR-708	0.333	4.618E-28	–0.000522	0.9905	0.167	0.0001741
E2F1^^^	miR-708	0.119	0.000132	0.13	0.002744	0.106	0.01712
CTBP2^*^	miR-708	–*0.19*	*8.999E-10*	0.0799	0.06715	–0.0134	0.7638
RAD21^^^	miR-708	0.206	2.502E-11	0.15	0.0005638	0.154	0.0005529
*C/EBP-β*^^^	miR-708	–*0.271*	*8.746E-19*	0.0728	0.09539	–*0.382*	*6.762E-19*
CTCF^*^	miR-708	0.16	2.434E-07	-0.0445	0.3086	0.0894	0.04538

TCGA mRNA/miRNA data showing correlation, and significance (*p* value) of miR-708-5p and various validated regulators of miR-708-5p expression in NSCLC (*n* = 864), LUAD (*n* = 442), and LUSC (*n* = 424) tumors. Italicized font indicates a significant negative correlation; underlined indicates significant positive correlation; and black font indicates no significant correlation. (^^^) represents a positive regulator of miR-708-5p expression, while (^*^) represents a repressor of miR- 708-5p expression.

**Table 2:** Mature miR-708-5p and p53 mRNA expression are positively correlated in NSCLC tumors

Subtype	Gene	miRNA	Correlation	Adj.R^2	*p* value
NSCLC	p53	miR-708	0.178	0.0306	9.66E-09
LUAD	p53	miR-708	0.107	0.00964	0.01374
LUSC	p53	miR-708	0.154	0.0219	0.0005222

TCGA mRNA/miRNA data showing correlation, adjusted R2, and significance (*p* value) of miR-708-5p and p53 mRNA in NSCLC (*n* = 864), LUAD (*n* = 442), and LUSC (*n* = 424) tumors. Underlined lettering indicates a significant positive correlation.

**Figure 2:**
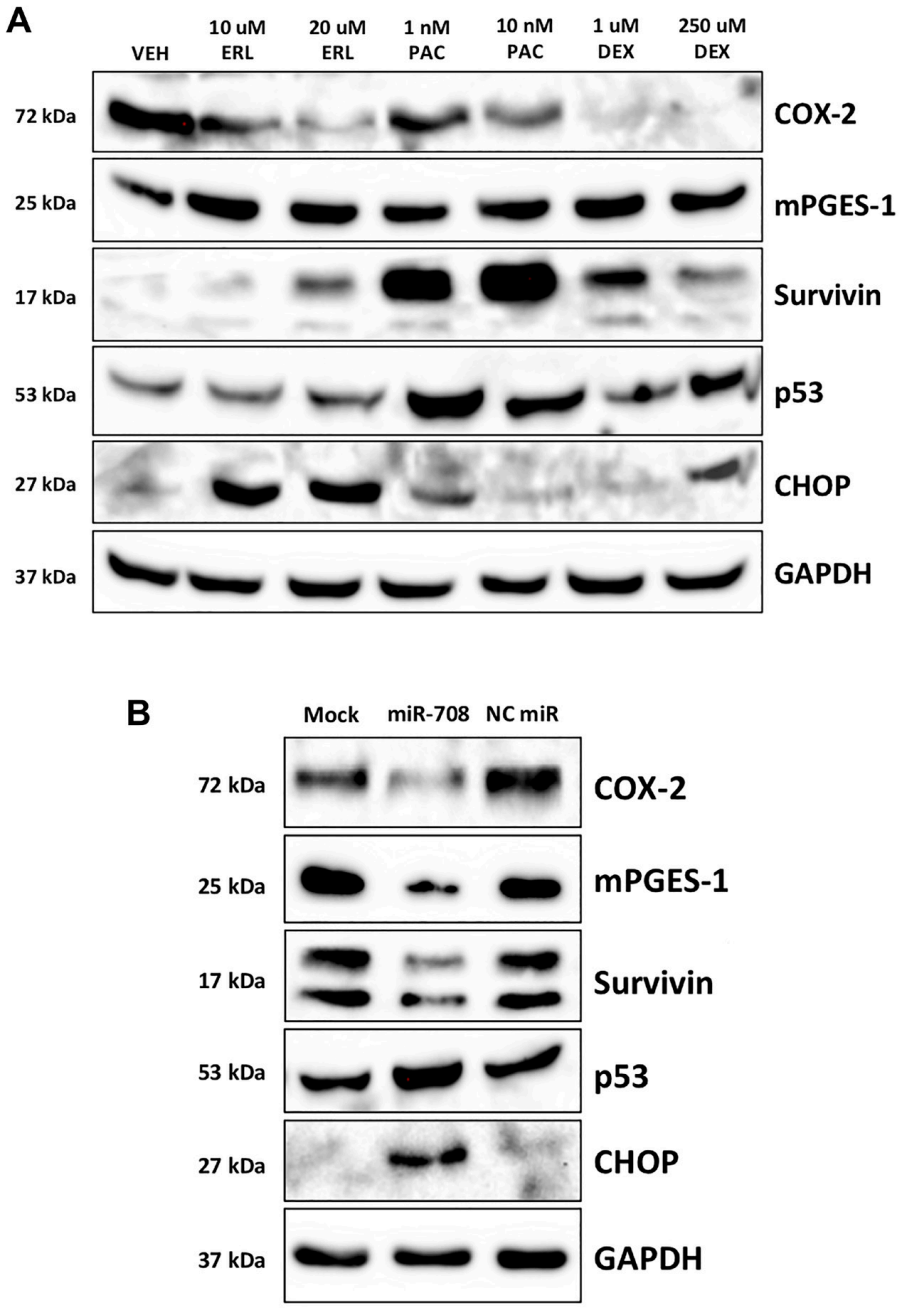
Chemotherapies and miR-708-5p induce survival-associated pathways in lung cancer cells. (**A**) Representative western blot depicting COX-2, mPGES-1, Survivin, p53, and CHOP protein expression in A549 cells treated with VEH, 10/20 uM ERL, 1/10 nM PAC, or 1/250 uM DEX for 24 hours. GAPDH served as a loading control. (**B**) Representative western blot analysis of COX-2, mPGES-1, Survivin, p53, and CHOP protein level in A549 cells treated with mock, 25 nM miR-708-5p, or 25 nM NC miR for 48 hours. GAPDH served as a loading control.

ERL treatment promoted high levels of CHOP, while PAC and DEX more modestly increased CHOP protein expression (Figure 2A). Moreover, high CHOP mRNA expression positively associated with increased survival rates in LUSC patients (Supplementary Figure 1, *p* = .014, *n* = 424), following similar patterns seen with miR-708-5p in NSCLC. We also observed that while ERL did not affect A549 p53 expression, PAC, and to a lesser extent DEX, induced p53 protein expression (Figure 2A). Next, we analyzed how miR-708-5p expression affected survival rates in p53 WT and mutant (MUT) tumors. miR-708-5p expression had no effect on survival in LUAD WT (Supplementary Figure 2A, *p* = 0.79, *n* = 240) or MUT (Supplementary Figure 2B, *p* = 0.45, *n* = 263) p53 tumors. While miR-708-5p levels in LUSC WT p53 tumors had no effect on survival (Supplementary Figure 2C, *p* = 0.91, *n* = 70), high miR-708-5p expression significantly enhanced survival in LUSC MUT p53 tumors (Supplementary Figure 2D, *p* = 0.041, *n* = 389). Collectively, these data suggest that high miR-708-5p may improve survival rates in LUSC patients containing p53 mutations.

### miR-708-5p enhances chemotherapeutic-induced expression of CHOP and p53 protein levels

**Figure 3:**
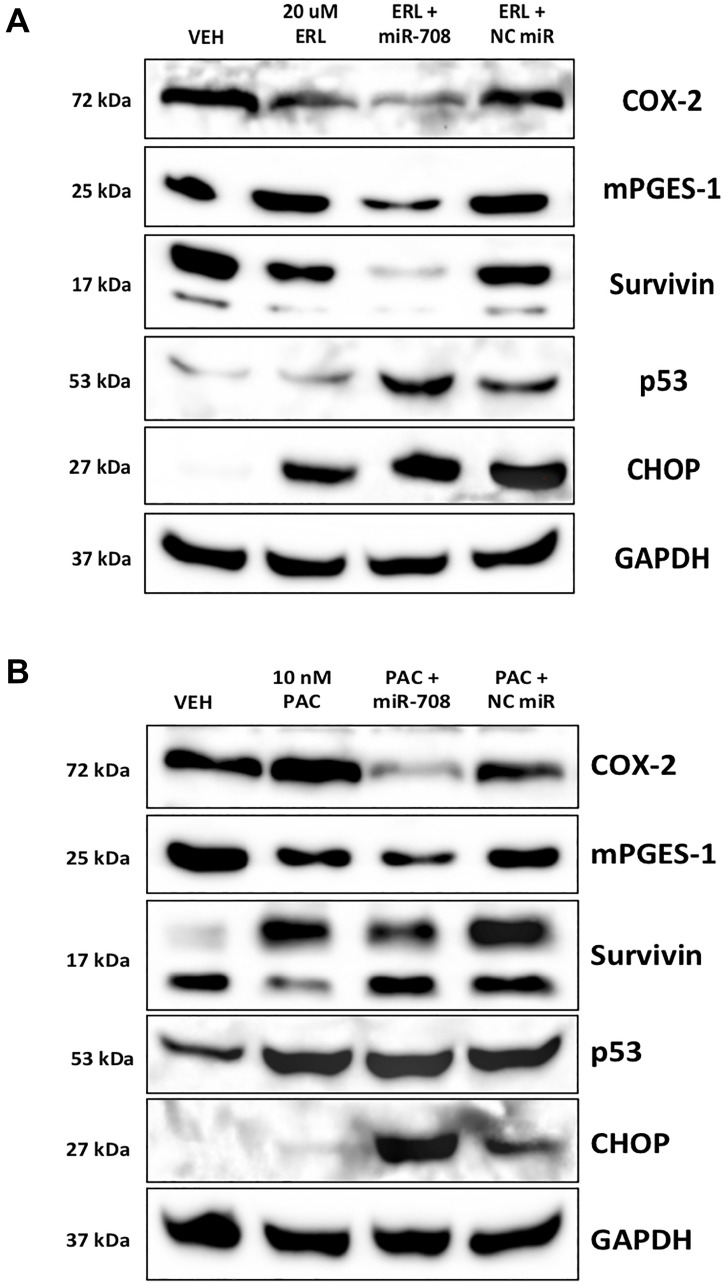
miR-708-5p enhances ERL/PAC regulation of AA pathway and apoptotic signaling expression in lung cancer cells. (**A**) Representative western blot analysis of COX-2, mPGES-1, Survivin, p53, and CHOP protein level in A549 cells treated with VEH, 20 uM ERL, 20 uM ERL + 25 nM miR-708-5p, or 20 uM ERL + 25 nM NC miR. GAPDH served as a loading control. (**B**) Representative western blot analysis of COX-2, mPGES-1, Survivin, p53, and CHOP protein level in A549 cells treated with VEH, 10 nM PAC, 10 nM PAC + 25 nM miR-708-5p, or 10 nM PAC + 25 nM NC miR. GAPDH served as a loading control.

Given the ability of ERL, PAC, and DEX to regulate miR-708-5p possibly through p53, and CHOP, we examined whether miR-708-5p regulates p53 and CHOP expression in lung cancer cells. To do this, we used A549 cells, as they are p53 WT. We found that A549 cells transiently transfected with miR-708-5p induced p53 and CHOP protein expression while simultaneously reducing COX-2, mPGES-1, and Survivin protein levels (Figure 2B). Given miR-708-5p’s ability to enhance p53 and CHOP protein expression, we examined the molecular and phenotypic consequences of combinatory miR-708-5p and chemotherapy treatments in lung cancer cells. First, we tested ERL alone or in combination with a NC miR/miR-708-5p in A549 cells. Western blot analysis revealed that ERL treatment alone decreased COX-2 protein expression while increasing CHOP protein levels (Figure 3A). ERL + miR-708-5p treatment further reduced COX-2 protein expression, while also suppressing mPGES-1 and Survivin protein levels (Figure 3A). CHOP expression was not enhanced further in the ERL + miR-708-5p samples, but ERL + miR-708-5p treatment increased p53 protein expression compared to vehicle, ERL, and ERL + NC miR samples (Figure 3A). Next, we investigated PAC treatment alone or in combination with a NC miR/miR-708-5p. We found that PAC had no effect on COX-2, mPGES-1, or CHOP protein expression, but induced Survivin and p53 protein levels (Figure 3B). Intriguingly, PAC + miR-708-5p combination treatment suppressed PAC-induced Survivin expression, while also reducing COX-2 and mPGES-1 protein levels (Figure 3B). While PAC + miR-708-5p treatment did not further increase p53 expression, the combination treatment strongly induced CHOP protein expression 10.2 fold in A549 cells (Figure 3B). Together, combination treatments of ERL/PAC + miR-708-5p suppress pro-tumorigenic signaling (COX-2, mPGES-1, Survivin) while activating pro-apoptotic pathways (CHOP, p53) greater than either therapy alone. Therefore, we further explored the phenotypic impact of combination treatments on lung cancer cells.

### miR-708-5p enhances ERL-induced cell cycle arrest and apoptosis in lung cancer cells

**Figure 4:**
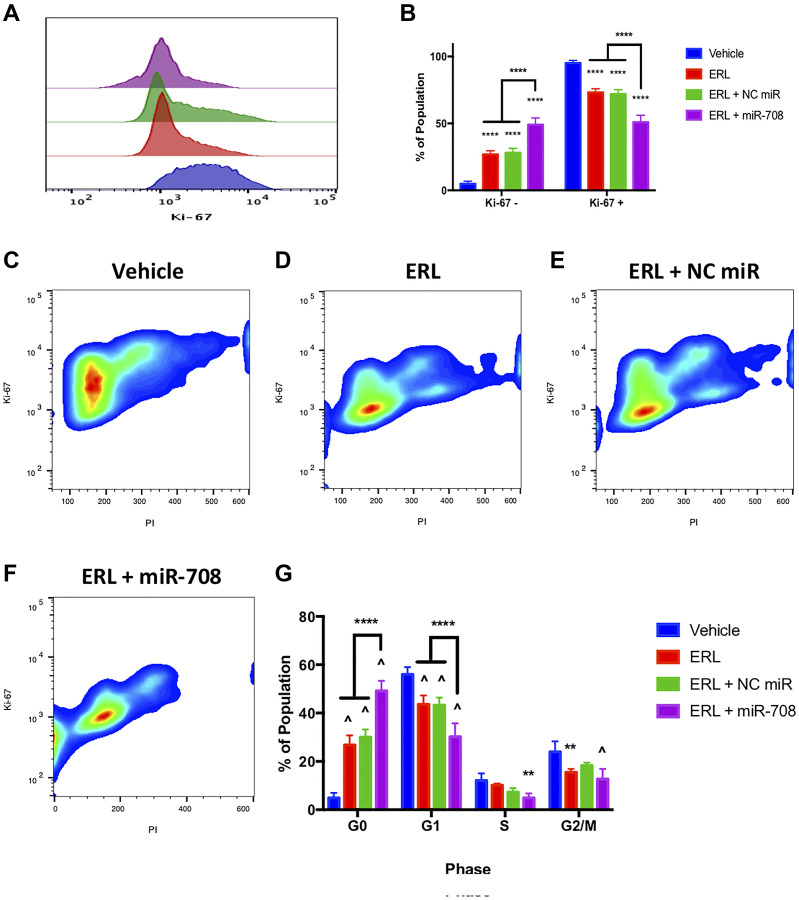
miR-708-5p enhances anti-proliferative effects of ERL in lung cancer cells. (**A**) Representative overlay histogram depicting Ki-67 positivity in A549 cells treated with vehicle (blue), 20 uM ERL (red), 20 uM ERL + 25 nM NC miR (green), or 20 uM ERL + 25 nM miR-708-5p (purple) for 48 hours. (**B**) Quantification of the Ki-67 negative (> 10^3^) and positive (< 10^3^) populations in various treatments from (A). (**C**–**F**) Representative smoothed graphs of flow cytometry data showing cell cycle stage based on Ki-67 (y-axis) and PI staining (x-axis) in A549 cells treated with (C) vehicle, (D) 20 uM ERL, (E) 20 uM ERL + 25 nM NC miR, or (F) 20 uM ERL + 25 nM miR-708-5p for 48 hours. Blue represents low cell area density, while red indicates high cell area density. (**G**) Quantification of cell cycle stage from (C–F). (^^^) indicates a significant difference (*p* < .0001) between vehicle and marked treatment. ^**^ *p* < . 01, ^****^ *p* < .0001, *n* ≥ 3.

To test the combinatory phenotypic effects of ERL/PAC and miR-708-5p in lung cancer cells, we examined changes in proliferation and apoptosis via Ki-67 and Annexin V staining, respectively. Ki-67 is a marker for proliferating cells, while Annexin V detects externalized phosphatidylserine (PS), a commonly used apoptosis marker [62, 63]. First, we examined whether ERL and miR-708-5p treatment enhanced anti-proliferative activities greater than either treatment alone. We found that ERL treatment alone significantly decreased A549 proliferating cells from 95% in vehicle treated samples to 73% in ERL treated samples (Figure 4, *p* < 0.0001, *n* ≥ 3). While ERL + NC miR treatment was similar to ERL treatment alone, ERL + miR-708-5p further suppressed lung cancer cell proliferation, with 51% of combinatory treated A549 cells were Ki-67+ (Figure 4, *p* < 0.0001, *n* ≥ 3). Next, we investigated how the combinatory treatment was altering lung cancer cell cycle progression. We discovered that ERL alone significantly reduced the percentage of A549 cells in G1 and G2/M phase (80% to 59%) while also inducing cells to accumulate in G0 phase (Figure 4, *p* < 0.01, *n* ≥ 3). Interestingly, ERL + miR-708-5p treatment further reduced the percent of A549 cells in G1 and G2/M phase to 43% and significantly reduced the number of cells in S phase by half (Figure 4, *p* < 0.01, *n* ≥ 3). This reduction in actively proliferating phases was paired with a significant accumulation of non-proliferation G0 phase A549 cells compared to ERL and ERL + NC miR treatments (Figure 4, *p* < 0.0001, *n* ≥ 3). Collectively, these data reveal that ERL and miR-708-5p cooperate to enhance anti-proliferative activities in lung cancer cells. Given these data, we investigated the effects this combinatory treatment had on apoptosis.

**Figure 5:**
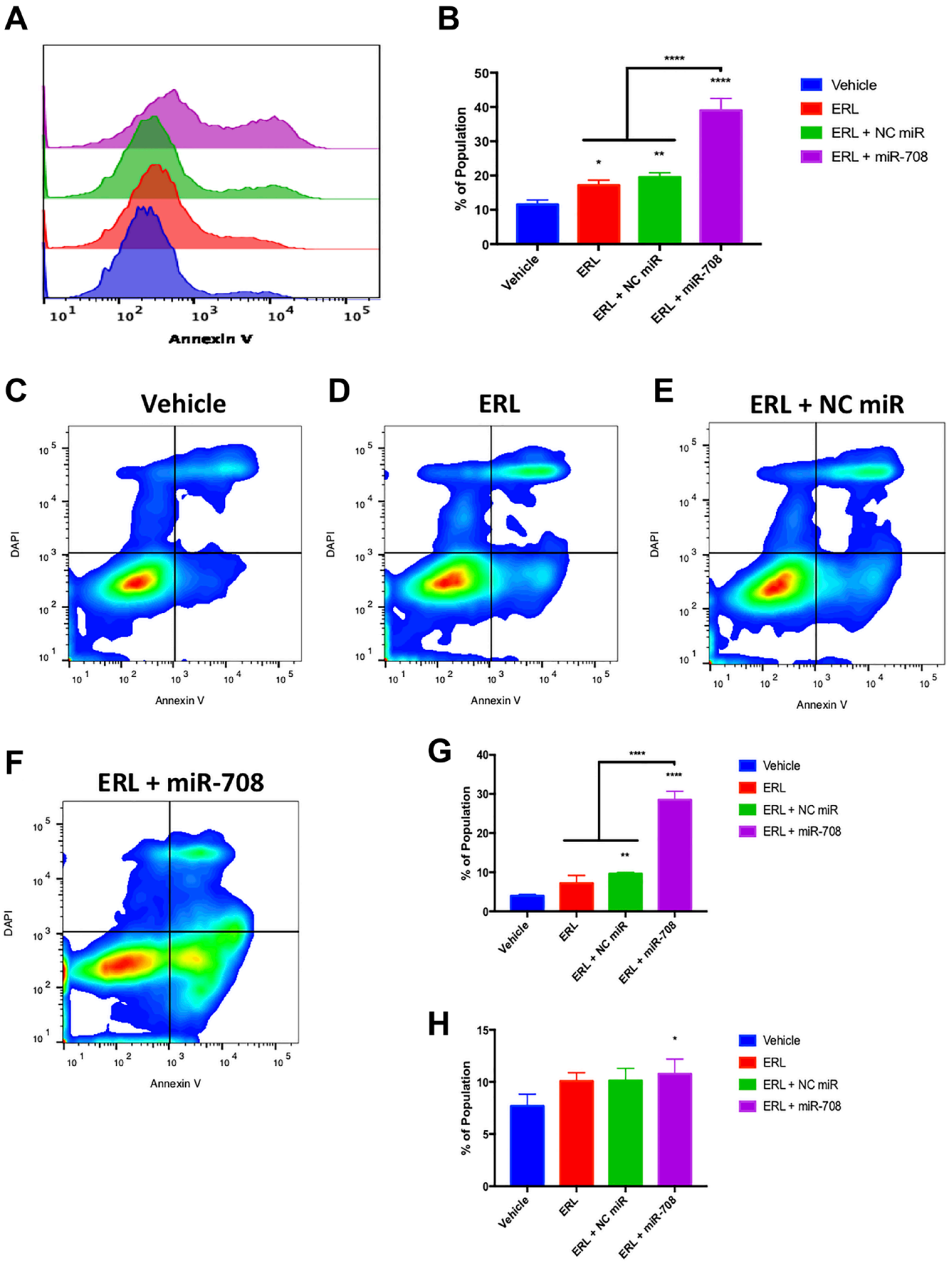
miR-708-5p amplifies ERL-induced apoptosis in lung cancer cells. (**A**) Representative overlay histogram of PS positivity in A549 cells treated with vehicle (blue), 20 uM ERL (red), 20 uM ERL + 25 nM NC miR (green), or 20 uM ERL + 25 nM miR-708-5p (purple) for 48 hours. (**B**) Quantification of PS positive (< 10^3.1^) populations from (A). (**C**–**F**) Representative smoothed graph flow cytometry data of A549 cells treated with (C) vehicle, (D) 20 uM ERL, (E) 20 uM ERL + 25 nM NC miR, or (F) 20 uM ERL + 25 nM miR-708-5p for 48 hours and stained with Annexin V and DAPI. (**G**) Quantification of the early apoptotic (Annexin V+, DAPI–) population from (C–F). (**H**) Quantification of the late apoptotic (Annexin V+, DAPI+) population from (C–F). ^*^ *p* < .05, ^**^ *p* < .01, ^****^ *p* < .0001, *n* ≥ 3.

While ERL and miR-708-5p have both been shown to induce apoptosis in lung cancer cells, researchers have not studied their combinatory potential. To examine this, we utilized flow cytometry and Annexin V staining. We observed that ERL significantly increased PS+ cell number compared to vehicle treatment (Figure 5, *p* < 0.05, *n* ≥ 3). ERL + miR-708-5p treatment enhanced the percentage of PS+ cells from 17% in ERL treatments to 39% in ERL + miR-708-5p treated samples (Figure 5, *p* < 0.0001, *n* ≥ 3). While these data reveal an increase in apoptosis, they do not distinguish between early and late apoptosis. As Figure 5E reveals, ERL + NC miR increased the percent of early apoptotic A549 cells (4% to 9.6%), while ERL + miR-708-5p treatment further intensified the early apoptotic population to 28% (Figure 5F and 5G, *p* < 0.05, *n* ≥ 3). Furthermore, ERL + miR-708-5p increased late apoptotic events while no other treatment was significantly different from our vehicle control (Figure 5F and 5H, *p* < 0.05, *n* ≥ 3). We conclude that while ERL induces apoptosis, ERL + miR-708-5p intensifies lung cancer cell death. These data, as well as the Ki-67 data, reveal an additive anti-tumor ERL and miR-708-5p combination therapy that reduces proliferation and survival greater than either treatment alone. Now that we have studied the combinatory effects of ERL and miR-708-5p, we repeated our studies with PAC and miR-708-5p.

### miR-708-5p enhances PAC-induced cell cycle arrest and apoptosis in lung cancer cells

**Figure 6:**
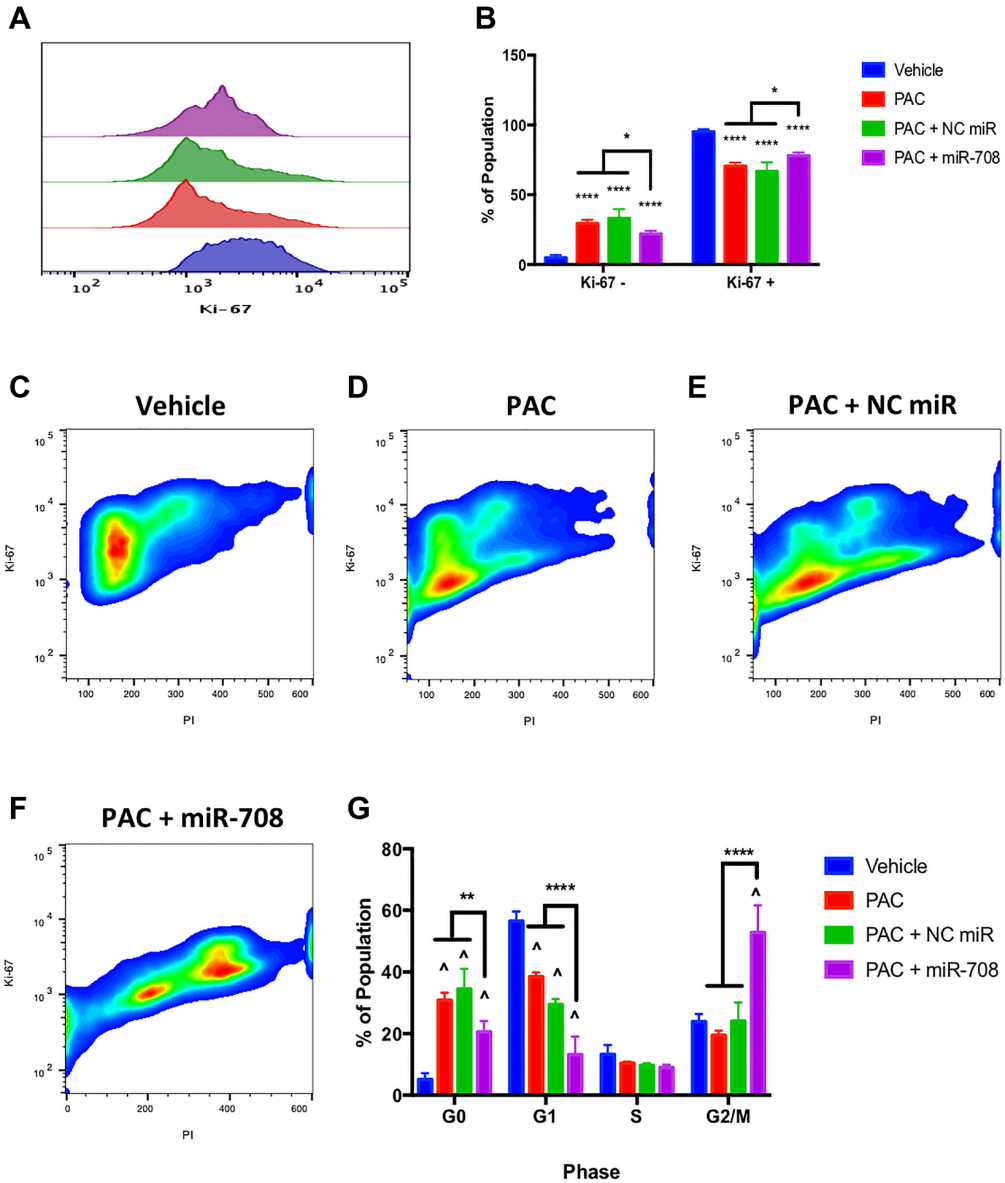
miR-708-5p enhances PAC-mediated anti-proliferative activities in lung cancer cells. (**A**) Representative overlay histogram depicting Ki-67 positivity in A549 cells treated with vehicle (blue), 10 nM PAC (red), 10 nM PAC + 25 nM NC miR (green), or 10 nM PAC + 25 nM miR-708- 5p (purple) for 48 hours. (**B**) Quantification of the Ki-67 negative (> 10^3^) and positive (< 10^3^) populations in various treatments from (A). (**C**–**F**) Representative smoothed graph of flow cytometry data showing cell cycle stage based on Ki-67 (y-axis) and PI staining (x-axis) in A549 cells treated with (C) vehicle, (D) 10 nM PAC, (E) 10 nM PAC + 25 nM NC miR, or (F) 10 nM PAC + 25 nM miR-708-5p for 48 hours. Blue represents low cell area density, while red indicates high cell area density. (**G**) Quantification of cell cycle stage from (C–F). (^) indicates a significant difference (*p* < .0001) between vehicle and marked treatment. ^**^ *p* < . 01, ^****^ *p* < .0001, *n* ≥ 3.

We investigated the effect PAC alone, or in combination with miR-708-5p, had on lung cancer proliferation. We found that PAC, PAC + NC miR, and PAC + miR-708-5p treatment significantly reduced the percent of Ki-67+ A549 cells (Figure 6A and 6B, *p* < 0.0001, *n* ≥ 3). Interestingly, while the PAC + miR-708-5p treatment decreased Ki-67 positivity, this treatment had a significantly higher Ki-67+ population compared to PAC and PAC + NC miR treated samples (Figure 6B, *p* < 0.05, *n* ≥ 3). Next, we examined how PAC and miR-708-5p were altering the cell cycle. We discovered that PAC + miR-708-5p treatment enhanced the percent of A549 cells in G0 to 20.6%, albeit significantly less than PAC (31%) and PAC + NC miR (35%) treatments (Figure 6, *p* < 0.01, *n* ≥ 3). PAC and PAC + NC miR treatments reduced the number of A549 cells in G1 phase from 56% to 39% and 29%, respectively, while increasing the percent of cells in G0 phase (Figure 6, *p* < 0.0001, *n* ≥ 3). Moreover, PAC + miR-708-5p further decreased the G1 population to 13%, while significantly increasing the percent of A549 cells in G2/M phase (Figure 6, *p* < 0.0001, *n* ≥ 3). While this may suggest the combination treatment is promoting proliferation, it does not take into account PAC’s anti-tumorigenic mechanism of action. PAC is a microtubule stabilizer that locks dividing cells in the G2/M phase. This PAC-induced stalling increases cellular stress, leading to apoptosis. Therefore, miR-708-5p enhanced the anti-proliferative effects of PAC, as it further decreased the percent of G1 cells while also enhancing the PAC’s G2/M-arresting effects (Figure 6, *p* < 0.0001, *n* ≥ 3). While PAC regulates proliferation, we also need to investigate the effects of PAC and miR-708-5p combination treatment on lung cancer cell apoptotic rates.

**Figure 7:**
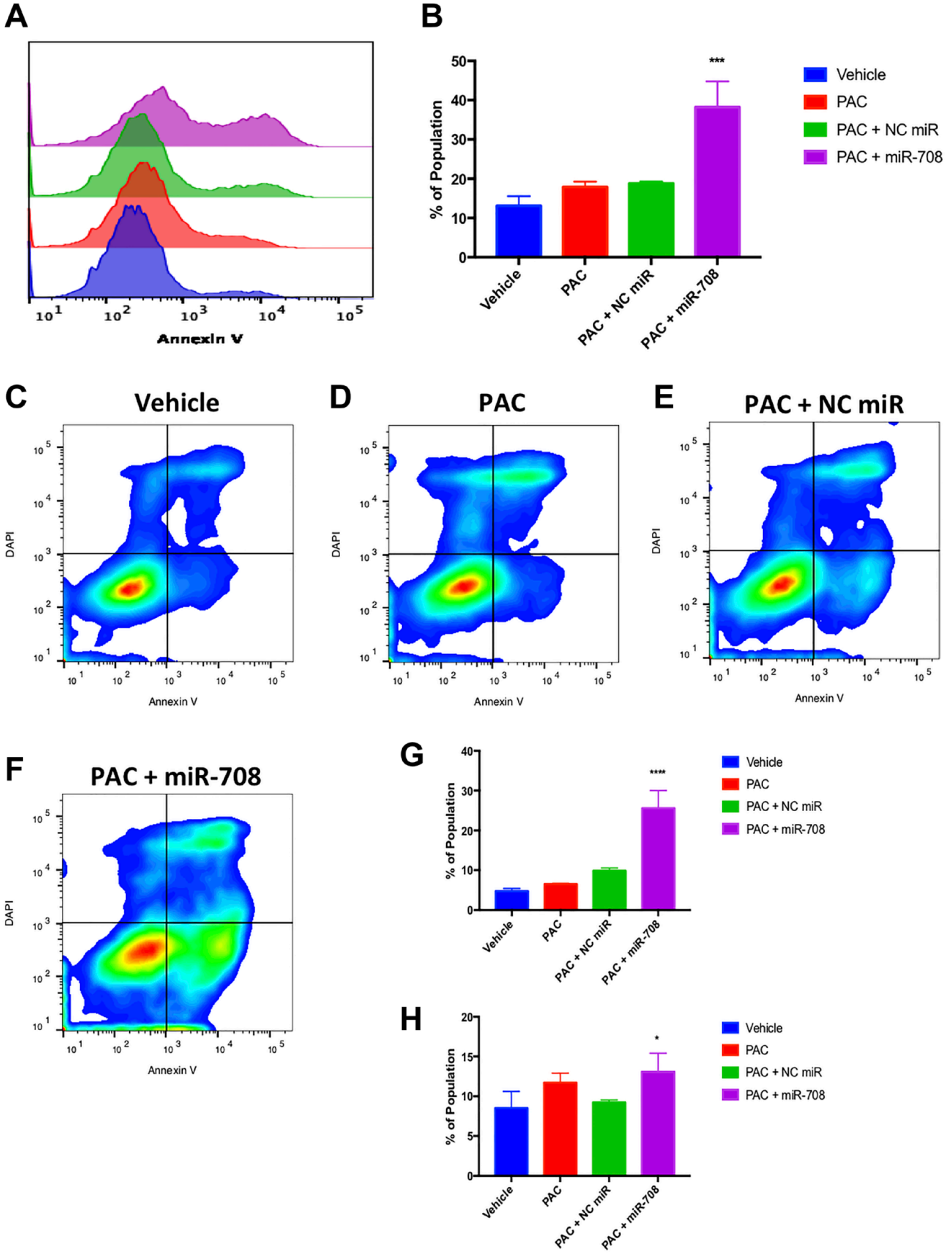
Combinatory miR-708-5p and PAC treatment induces apoptosis in lung cancer cells. (**A**) Representative overlay histogram of PS positivity in A549 cells treated with vehicle (blue), 10 nM PAC (red), 10 nM PAC + 25 nM NC miR (green), or 10 nM PAC + 25 nM miR-708-5p (purple) for 48 hours. (**B**) Quantification of PS positive (< 10^3.1^) populations from (A). (**C**–**F**) Representative smoothed graph flow cytometry data of A549 cells treated with (C) vehicle, (D) 10 nM PAC, (E) 10 nM PAC + 25 nM NC miR, or (F) 10 nM PAC + 25 nM miR-708-5p for 48 hours and stained with Annexin V and DAPI. (**G**) Quantification of the early apoptotic (Annexin V+, DAPI–) population from (C–F). (**H**) Quantification of the late apoptotic (Annexin V+, DAPI+) population from (C–F). ^*^ *p* < .05, ^****^ *p* < .0001, *n* ≥ 3.

Given PAC’s anti-tumor characteristics, as well as miR-708-5p’s pro-apoptotic functions, we studied the combinatory effects of these two treatments in lung cancer cells. PAC treatment alone increased the number of late apoptotic cells compared to vehicle control, while there was no significant increase in PS positivity or early apoptotic events (Figure 7, *p* < 0.05, *n* ≥ 3). PAC + NC miR did not significantly affect PS positivity, early, or late apoptotic events when compared to vehicle control (Figure 7, *p* = n.s, *n* ≥ 3). Conversely, combination PAC + miR-708-5p dramatically increased the percent of PS+ A549 cells from 11.5% to 39% (Figure 7, *p* < 0.0001, *n* ≥ 3). Moreover, PAC + miR-708-5p treatment increased the number of early and late apoptotic/dead cells compared to vehicle control (Figure 7, *p* < .05, *n* ≥ 3). Collectively, these data suggest that PAC + miR-708-5p treatment significantly enhances the pro-apoptotic effects of PAC on lung cancer cells. These data paired with our proliferation data form the basis for exploring the therapeutic combinatory potential of PAC and miR-708-5p in lung cancer.

### miR-708-5p is expressed lower and non-responsive to ERL/PAC treatment in chemoresistant lung cancer cells

**Figure 8:**
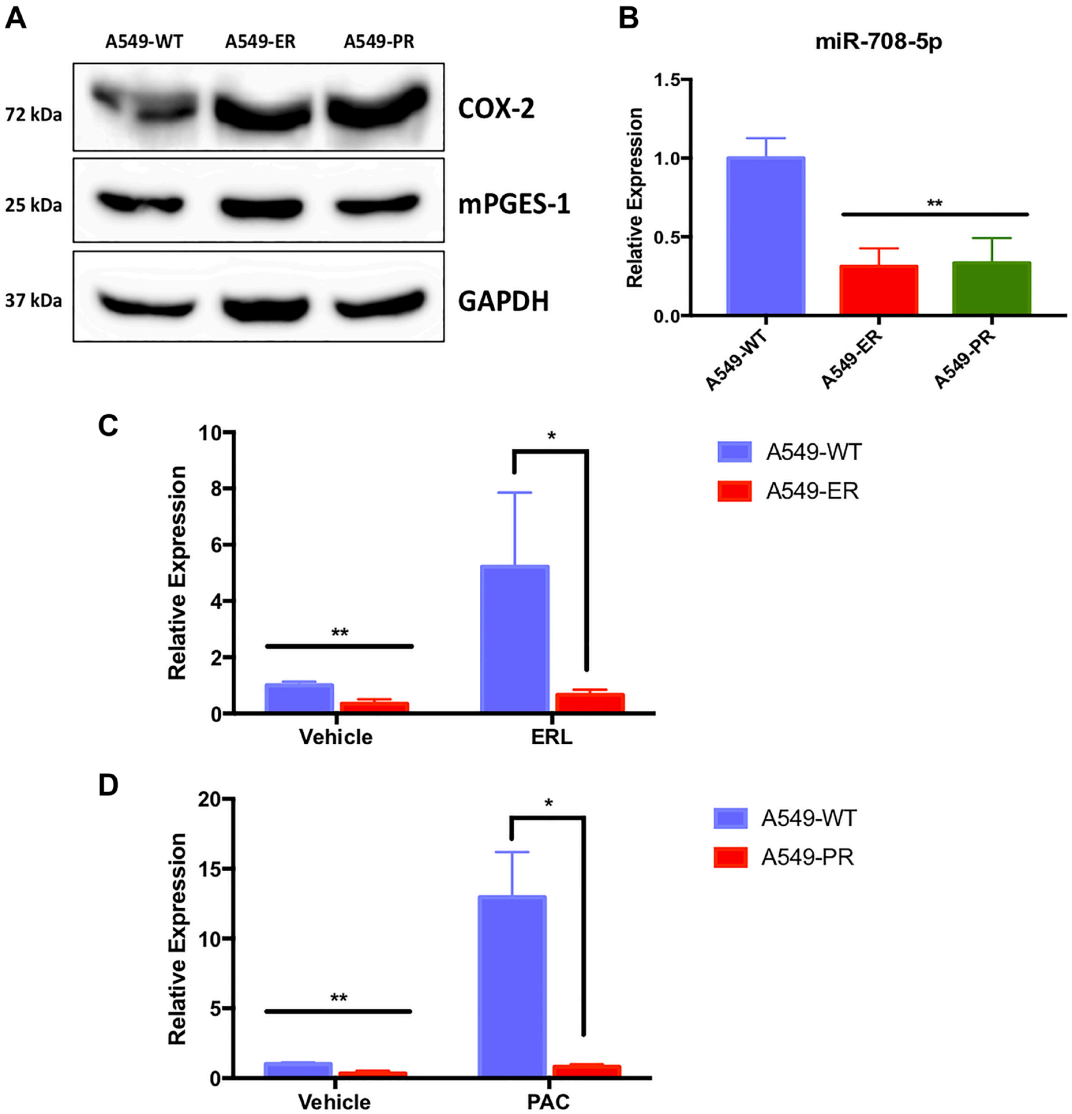
A549-ER and A549-PR cells have altered miR-708-5p and AA pathway expression. (**A**) Representative western blot of COX-2 and mPGES-1 baseline protein expression in A549-WT, A549-ER, and A549-PR cells. GAPDH served as a loading control. (**B**) RT-qPCR of baseline mature miR-708-5p in A549-WT (blue), A549-ER (red), and A549-PR (green) cells. (B) Representative western blot of COX-2 and mPGES-1 baseline protein expression in A549-WT, A549-ER, and A549-PR cells. GAPDH served as a loading control. (**C**) RT-qPCR of mature miR-708-5p in A549-WT (blue) and A549-ER (red) cells treated with vehicle or 20 uM ERL for 24 hours. (**D**) RT-qPCR of mature miR-708-5p in A549-WT (blue) and A549-PR (red) cells treated with vehicle or 10 nM PAC for 24 hours. miR-708-5p expression was normalized to miR-15a and analyzed using the 2-ΔΔCT method. ^*^ *p* < .05, ^**^ *p* < .01, *n* ≥ 3.

While ERL and PAC are commonly used to treat lung tumors, their efficacy in the clinic is limited because of developed resistance to these drugs. Given the annotated functions of COX-2/mPGES-1 derived PGE_2_ and miR-708-5p in resistance, we investigated the role of AA signaling and miR-708-5p in ERL and PAC resistance. First, we created A549 ERL resistant (A549-ER) and PAC resistant (A549-PR) cell lines as previously described [64]. We confirmed our cells were resistant by comparing chemotherapeutic-induced changes in proliferation and apoptosis in naïve (A549-WT) and resistant (A549-ER, A549-PR) cell lines (Supplementary Figures 3–6). We found that COX-2 protein expression was higher in our A549-ER and A549-PR cells compared to A549-WT cells (Figure 8A). Next, we found that miR-708-5p expression was significantly lower at baseline in A549-ER (–69%) and A549-PR (–66%) cells compared to A549-WT cells (Figure 8B, *p* < .01, *n* = 3). We also examined the ability of ERL and PAC to induce miR-708-5p in our resistant cell lines. Beyond being expressed less in the resistant cell lines, miR-708-5p expression was no longer responsive to ERL (Figure 8C, *p* < .05, *n* = 3) or PAC (Figure 8D, *p* < .05, *n* = 3) treatment in resistant cells, respectively. As miR-708-5p is underexpressed and no longer responsive to ERL or PAC treatments in our resistant cells, we explored the phenotypic value of miR-708-5p treatment to overcome resistance in our lung cells.

### miR-708-5p partially restores ERL’s anti-proliferative and pro-apoptotic effects in chemoresistant lung cancer cells

**Figure 9:**
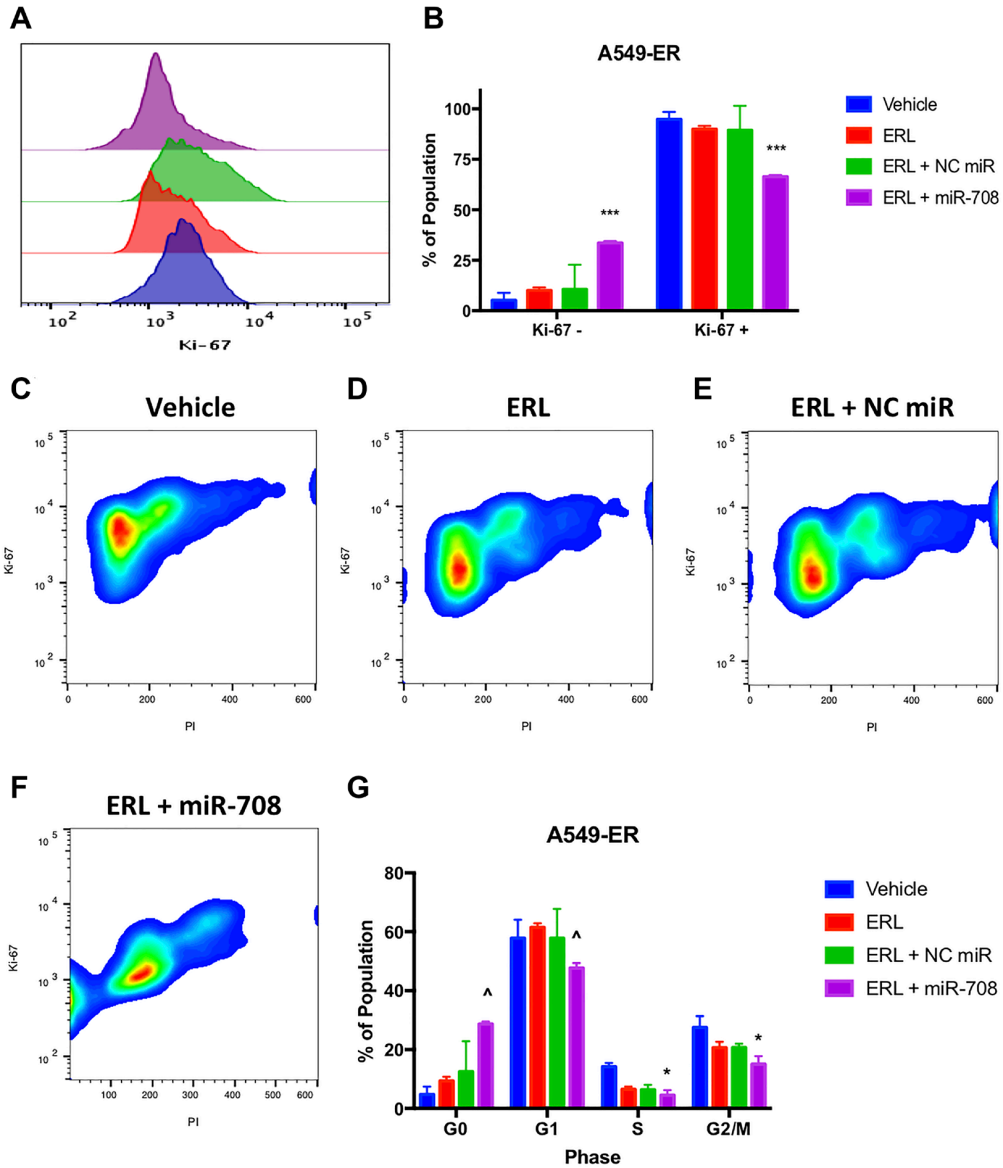
miR-708-5p + ERL reduces Ki-67+, G0 → **G1 transition, S, and G2/M phase in A549-ER cells.** (**A**) Representative overlay histogram depicting the number of A549-ER cells that were Ki-67 negative (> 10^3^) and positive (< 10^3^) as measured by flow cytometry. For this figure, sample colors are as followed: vehicle (blue), 20 uM ERL (red), 20 uM ERL + 25 nM NC miR (green), and 20 uM ERL + 25 nM miR- 708-5p (purple). (**B**) Quantification of the +/– Ki-67 populations in various treatments from (A). (**C**–**F**) Representative cell cycle stage graphs of (C) vehicle, (D) 20 uM ERL, (E) 20 uM ERL + 25 nM NC miR, or (F) 20 uM ERL + 25 nM miR-708-5p treated A549-ER cells evaluated by flow cytometry. Representative smoothed graph showing cell cycle stage based on Ki-67 (y-axis) and PI staining (x-axis). Blue represents low cell area density, while red indicates high cell area density. Boxes identify populations as followed: G0 is -Ki- 67/low PI, G1 is +Ki-67/low PI, S is +Ki-67/Intermediate PI, G2/M is +Ki-67/High PI. (**G**) Quantification cell cycle stage of graphs from (C–F). ^*^ *p* < .05, ^***^ *p* < .001, *n* ≥ 3, ^^^ *p* < .0001, *n* ≥ 3.

**Figure 10:**
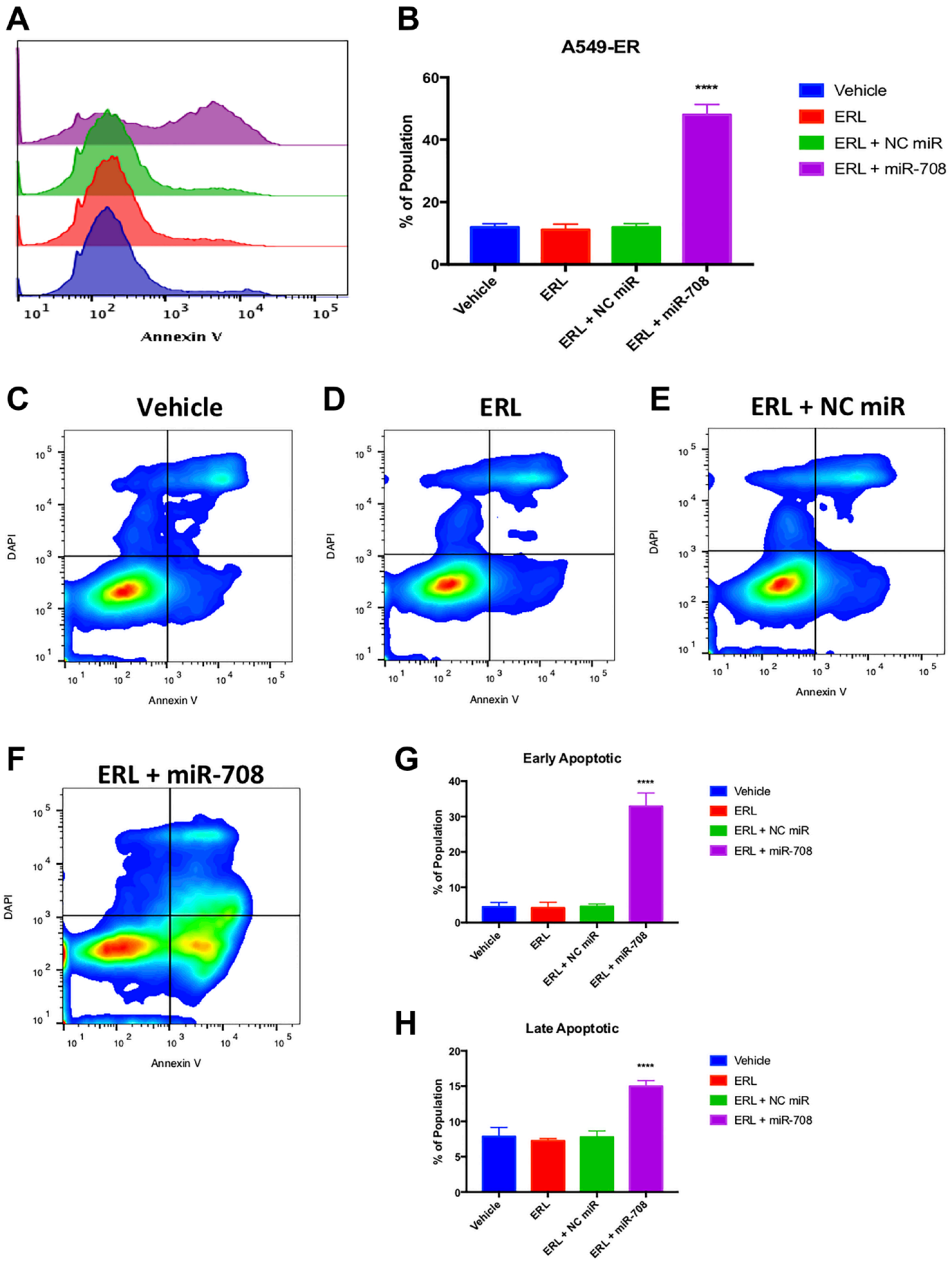
Combinatory miR-708-5p + ERL treatment induces apoptosis in A549-ER cells. (**A**) Representative overlay histogram of PS (Annexin V) negative (> 10^3^) and positive (< 10^3^) A549-ER populations as measured by flow cytometry. For this figure, sample colors are as followed: vehicle (blue), 20 uM ERL (red), 20 uM ERL + 25 nM NC miR (green), and 20 uM ERL + 25 nM miR-708-5p (purple). (**B**) Quantification of PS positive (< 103.1) populations from (A). (**C**–**F**) Representative smoothed graph flow cytometry data of A549-ER cells treated with (C) vehicle, (D) 20 uM ERL, (E) 20 uM ERL + 25 nM NC miR, or (F) 20 uM ERL + 25 nM miR-708-5p for 48 hours and stained with Annexin V and DAPI. (**G**) Quantification of the early apoptotic (Annexin V+, DAPI-) population from (C–F). (**H**) Quantification of the late apoptotic (Annexin V+, DAPI+) population from (C–F). ^****^ *p* < .0001, *n* ≥ 3.

Given enhanced AA signaling paired with decreased miR-708-5p expression in our resistant cell lines, we explored the ability of miR-708-5p to resensitize A549-ER and A549-PR cells to ERL and PAC treatments, respectively. While ERL treatment alone or in combination with NC miR did not affect Ki-67 positivity in A549-ER cells, ERL + miR-708-5p decreased A549-ER Ki-67+ cell number by 24% (Figure 9, *p* < .001, *n* ≥ 3). ERL and ERL + NC miR treatments insignificantly reduced S and G2/M populations in A549-ER cells, whereas ERL + miR-708-5p significantly suppressed the number of cells in S and G2/M phase by 21% (Figure 9, *p* < .05, *n* ≥ 3). Moreover, ERL and ERL + NC miR had no effect on G0 and G1 populations, but ERL + miR-708-5p treatment significantly decreased the G1 population by 20% in A549-ER cells (Figure 9, *p* < .0001, *n* ≥ 3). Next, we investigated survival rates in A549-ER cells. We found that ERL and ERL + NC miR treatments had no effect on PS+ (Figure 10, *p* = n.s., *n* ≥ 3) or apoptosis rates (Figure 10, *p* = n.s., *n* ≥ 3) compared to vehicle control. On the other hand, ERL + miR-708-5p significantly increased the number of PS+ cells from 12% in vehicle samples to 48% in ERL + miR-708-5p samples (Figure 10A and 10B, *p* < .0001, *n* ≥ 3). ERL + miR-708-5p treatment significantly elevated the percent of early apoptotic and late apoptotic/dead cells compared to vehicle, ERL, and ERL + NC miR treatments (Figure 10F–10H, *p* < .0001, *n* ≥ 3). Collectively, these data suggest that ERL + miR-708-5p represses proliferation and stimulates apoptosis in ERL resistant lung cancer cells. After we examined ERL and miR-708-5p’s effects on A549-ER cells, we also replicated our studies in A549-PR cells.

### miR-708-5p partially restores PAC’s anti-proliferative and pro-apoptotic effects in chemoresistant lung cancer cells

**Figure 11:**
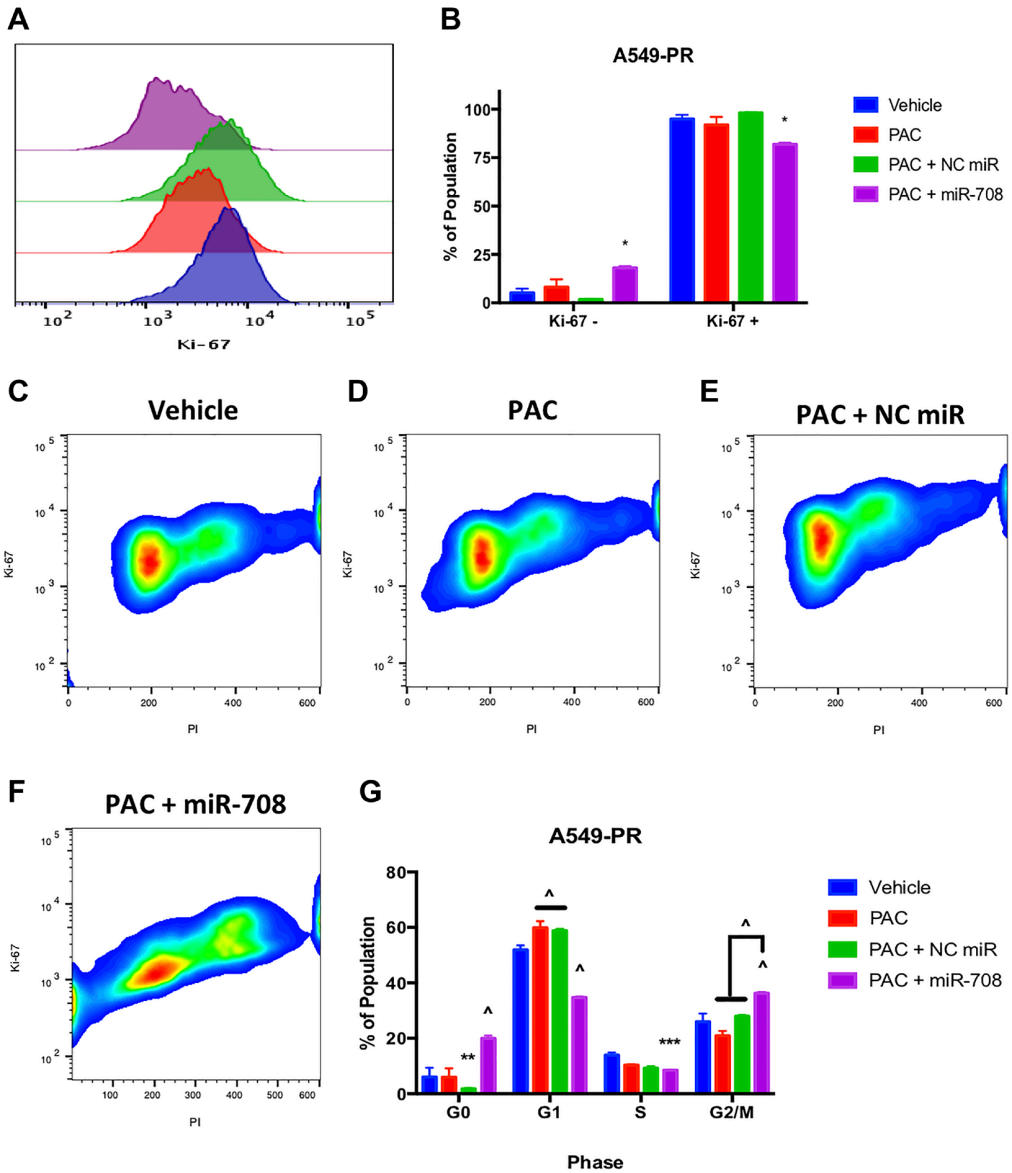
miR-708-5p + PAC reduces Ki-67+, G0 → **G1 transition, S, and G/2M phase in A549-PR cells.** (**A**) Representative overlay histogram depicting Ki-67 positivity in A549-PR cells treated with vehicle (blue), 10 nM PAC (red), 10 nM PAC + 25 nM NC miR (green), or 10 nM PAC + 25 nM miR-708- 5p (purple) for 48 hours. (**B**) Quantification of the Ki-67 negative (>10^3^) and positive (<10^3^) populations in various treatments from (A). (**C**–**F**) Representative smoothed graph of flow cytometry data showing cell cycle stage based on Ki-67 (y-axis) and PI staining (x-axis) in A549-PR cells treated with (C) vehicle, (D) 10 nM PAC, (E) 10 nM PAC + 25 nM NC miR, or (F) 10 nM PAC + 25 nM miR-708-5p for 48 hours. Blue represents low cell area density, while red indicates high cell area density. (**G**) Quantification of cell cycle stage from (C–F). (^) indicates a significant difference (*p* < .0001) between vehicle and marked treatment. ^*^ *p* < .05, ^**^ *p* < . 01, ^****^ *p* < .0001, *n* ≥ 3.

**Figure 12:**
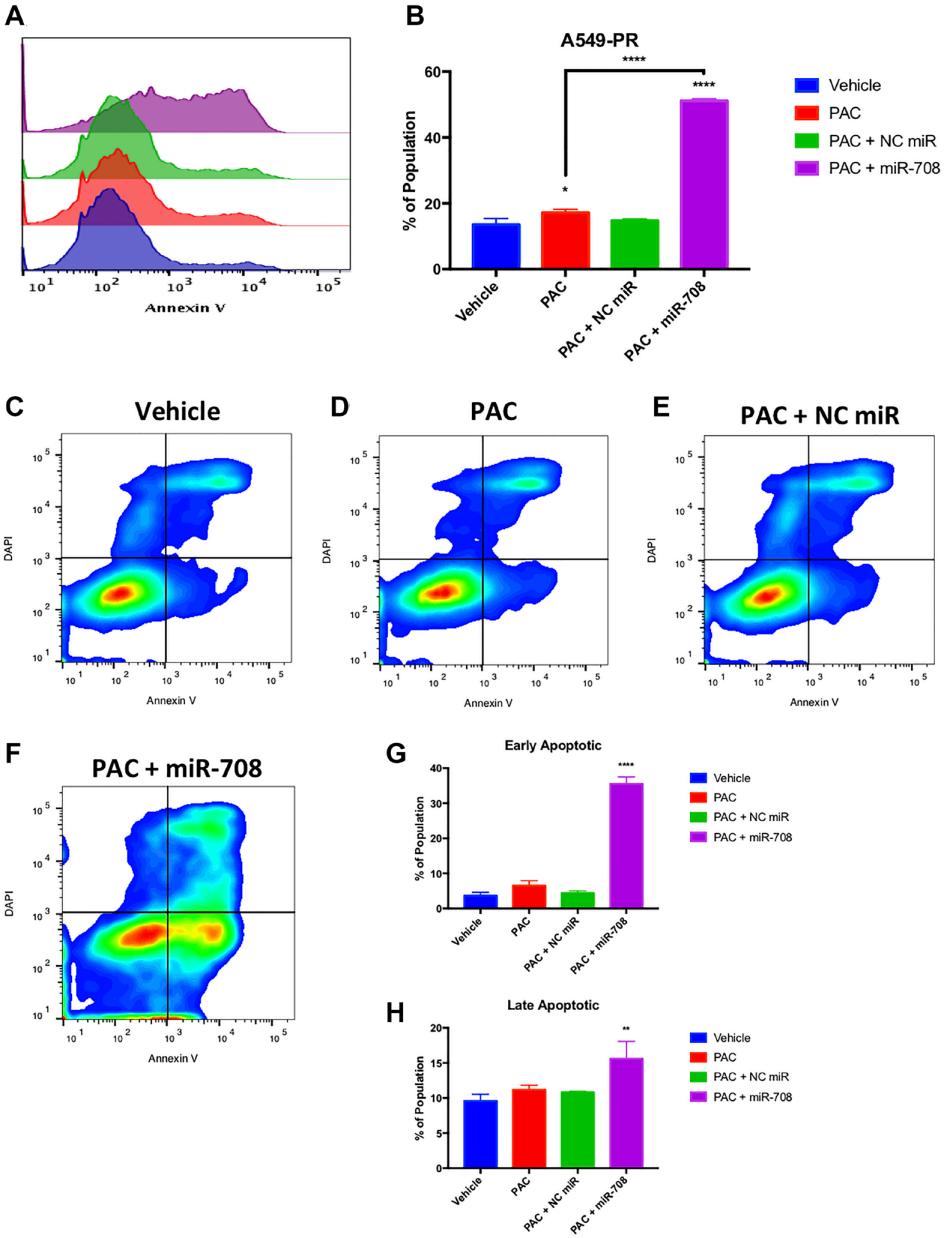
Combinatory miR-708-5p + PAC treatment induces apoptosis in A549-PR cells. (**A**) Representative overlay histogram of PS (Annexin V) negative (> 10^3^) and positive (< 10^3^) A549-PR populations as measured by flow cytometry. For this figure, sample colors are as followed: vehicle (blue), 10 nM PAC (red), 10 nM PAC + 25 nM NC miR (green), and 10 nM PAC + 25 nM miR-708-5p (purple). (**B**) Quantification of PS+ populations from (A). (**C**–**F**) Representative smoothed graph flow cytometry data of A549-PR cells treated with (C) vehicle, (D) 10 nM PAC, (E) 10 nM PAC + 25 nM NC miR, or (F) 10 nM PAC + 25 nM miR-708-5p for 48 hours and stained with Annexin V and DAPI. (**G**) Quantification of the early apoptotic (Annexin V+, DAPI–) population from (C–F). (**H**) Quantification of the late apoptotic (Annexin V+, DAPI+) population from (C–F). ^*^ *p* < .05, ^**^ *p* < .01, ^****^ *p* < .0001, *n* ≥ 3.

We first measured PAC induced changes in A549-PR proliferation. PAC alone or in combination with a NC miR had no effect on the number of Ki-67+ cells compared to vehicle control (Figure 11, *p* = n.s., *n* ≥ 3). Conversely, combination treatment of PAC + miR-708-5p significantly reduced the percent of proliferating A549-PR cells from 95 to 82% (Figure 11A and 11B, *p* < .05, *n* ≥ 3). More specifically, it appears PAC + miR-708-5p is reducing proliferation by driving A549-PR cells into G0 phase, as well as reducing the percent of cells in S phase by 5.4% (Figure 11, *p* < .001, *n* ≥ 3). PAC alone or in combination with NC miR had no affect the percentage of A549-PR cells in S or G2/M phases (Figure 11, *p* = n.s., *n* ≥ 3). Lastly, PAC + miR-708-5p significantly increased the G2/M A549-PR population by 10–16% compared to other treatments (Figure 11, *p* < .0001, *n* ≥ 3). As previously stated, PAC stalls proliferating cells in G2/M phase by stabilizing microtubules. This prevents cell division, which leads to increased cellular stress and apoptosis. Therefore, while it appears PAC may be promoting cell division, it is actually locking cells into G2/M phase, which ultimately leads to apoptosis. Therefore, we examined if PAC + miR-708-5p treatment modulated apoptotic rates in A549-PR cells. We discovered that PAC alone increased the PS+ population from 13% to 17%, which was further increased in the PAC + miR-708-5p co-treatment to 51% (Figure 12, *p* < .05, *n* ≥ 3). Moreover, it appears that PAC + miR-708-5p treatment is amplifying both early apoptotic and late apoptotic/dead events compared to other treatments in A549-PR cells (Figure 12, *p* < .01, *n* ≥ 3). Together, these results suggest miR-708-5p may be an important component of PAC resistance in lung cancer cells. Co-treatment of PAC + miR-708-5p helps to overcome resistance, highlighting the therapeutic potential of miR-708-5p in naïve and chemotherapeutic-resistant lung tumors.

## DISCUSSION

Lung cancer is a collection of tumors arising from varying cell types within the lung. While late detection is a contributing factor to effectively treating lung cancer, the 5-year survival rate amongst Stage I-II NSCLC patients is still less than 60% [65]. As most patients are not diagnosed until late-stage disease, overall 5-year NSCLC survival rates are 23% [65]. These data highlight the fact that lung cancer therapies are often ineffective at treating patients regardless of disease stage. While surgery and radiotherapy are utilized when possible, the vast majority of NSCLC patients undergo combinatory chemotherapeutic treatment. These drugs effectively reduce tumor growth initially, yet many patients discontinue treatment due to toxicity and resistance. Researchers have identified multiple signaling pathways involved in lung cancer chemoresistance, including COX-2/mPGES-1 derived PGE_2_. While COX-2 inhibitors have shown promise in the clinic, their use is limited due to severe side effects. Therefore, it is crucial to develop novel therapeutics that comprehensively suppress tumor growth without generating severe side effects.

In this communication, we identify a novel miRNA, miR-708-5p, that is regulated by chemotherapies in NSCLC cells (Figure 1). Moreover, combinatory treatments of chemotherapies and miR-708-5p suppress lung cancer cell proliferation and survival greater than either treatment alone (Figures 4–7). Mechanistically, we discovered that chemotherapies suppressed AA pathway expression while inducing miR-708-5p in lung cancer cells (Figure 1). We identified p53 and CHOP as potential transcription factors regulating chemotherapeutic-induced miR-708-5p expression (Tables 1 and 2, Figures 2 and 3). Interestingly, miR-708-5p also induced p53 and CHOP protein expression, suggesting a novel positive feedback loop (Figure 2). Next, we created lung cancer cells that were resistant to erlotinib (ERL, A549-ER) and paclitaxel (PAC, A549-PR). We found that COX-2 expression was greater and miR-708-5p lower in our resistant cell lines compared with chemosensitive lung cancer cells (A549-WT, Figure 8). ERL and PAC treatments also no longer induced miR-708-5p expression in resistant cell lines (Figure 8). Additionally, chemotherapies alone did not induce apoptosis or decrease proliferation in A549-ER/PR cells (Figure 8). Addition of miR-708-5p did however restore chemosensitivity and anti-tumor effects to our ERL and PAC resistant cells (Figures 9–12). Collectively, our data reveal a potent synergy between chemotherapies and miR-708-5p in suppressing lung cancer cell proliferation and survival. This work provides the foundation for studying the therapeutic value of miR-708-5p *in vivo* in combination with frontline chemotherapies in lung cancer. Although we present a novel enhancement in anti-tumor activity between chemotherapies and miR-708-5p, many questions remain.

First, we must decipher the most efficacious combinatory cancer treatments with miR-708-5p. This should focus first on testing the ability of miR-708-5p to improve outcomes in combination with the standard of care in various lung cancer subtypes. We focused on widely used therapies (ERL/PAC) with which COX-2 has been implicated in regulating resistance. Therefore, miR-708-5p targeting of COX-2 may prevent and/or overcome ERL and PAC resistance. Expanding this approach to other frontline chemotherapies would provide crucial data for understanding to which therapies miR-708-5p may add value. As chemotherapies’ mechanisms of action are well studied, investigators may recognize pro-resistance pathways common across different treatments. Therefore, researchers would also benefit from transcriptomic and proteomic studies testing the global effects of various chemotherapies in lung cancer cells. This may identify novel miR-708-5p targets as well as regulators of miR-708-5p expression.

One of the largest gaps in knowledge is identifying how miR-708-5p is adding to the anti-tumor activities of ERL and PAC. Both chemotherapies have distinct tumor suppressive mechanisms, yet miR-708-5p is involved in both. Therefore, it is likely that miR-708-5p is targeting genes responsible for promoting resistance and survival. We have identified several pro-survival genes involved in resistance that miR-708-5p is targeting in our lung cancer cells. First, we have previously shown that miR-708-5p targets COX-2 and mPGES-1 expression in lung cancer cells [55]. In this study, we show that while ERL and PAC can partially suppress COX-2 expression, miR-708-5p further represses COX-2 protein levels while also reducing mPGES-1 protein expression (Figure 3). We also examined changes in the expression of the miR-708-5p target Survivin after ERL/PAC treatment, as it is a prominent pro-survival protein implicated in resistance [66]. ERL does not alter Survivin levels, and PAC enhances Survivin expression (Figure 1). Interestingly, miR-708-5p co-treatment with either chemotherapy resulted in a pronounced reduction in Survivin protein levels (Figure 3). This combinatory inhibition of primary oncogenic drivers with chemotherapies as well as compensatory signaling with miR-708-5p appears to be highly effective at reducing lung cancer cell proliferation while enhancing cell death. COX-2 protein expression was also higher in our ERL/PAC resistant lung cancer cells compared to naïve lung cancer cells (Figure 8). Furthermore, miR-708-5p resensitized resistant cells to their respective chemotherapy (Figures 9–12). Collectively, these data suggest that miR-708-5p may act dually in resistance by preventing as well as overcoming acquired resistance in lung cancer cells. Given miR-708-5p’s targeting of numerous pro-survival genes, it is likely COX-2, mPGES-1, and Survivin repression is only partly responsible for the tumor suppressive changes we have seen in lung cancer cells. While we have identified several miR-708-5p targets possibly involved in chemoresistance, we did have not investigated pro-apoptotic pathways miR-708-5p may be activating.

While we have not fully uncovered the miR-708-5p-mediated molecular mechanisms in resistance, we have identified several key apoptosis regulators miR-708-5p may be coordinating. Figure 2 reveals that miR-708-5p alone can induce both CHOP and p53 protein expression. Additionally, combination treatment of ERL/PAC and miR-708-5p amplified CHOP and p53 protein expression greater than either treatment alone (Figure 3). How miR-708-5p is inducing CHOP and p53 expression remains enigmatic. It is most likely that miR-708-5p is indirectly increasing their expression by repressing pro-survival signaling. This increased cellular stress activates stress response proteins such as CHOP and p53, which amplify pro-apoptotic signaling. Interestingly, there may be a positive feedback loop between CHOP, p53, and miR-708-5p, as ERL and PAC both enhance all three gene’s expression. Given previous studies showing that CHOP stimulates miR-708-5p expression, it likely that CHOP is responsible for ERL-induced miR-708-5p expression in lung cancer cells. There also may be other stress-associated transcription factors promoting miR-708-5p expression. For example, PAC activates different pro-apoptotic signaling mechanisms than ERL. CHOP alone cannot account for miR-708-5p expression changes in PAC treated lung cancer cells, as CHOP protein levels are modestly altered by PAC treatment. While we speculate that p53 may be a miR-708-5p transcriptional regulator, we cannot make any definitive conclusions. miR-708-5p and p53 mRNA expression were positively correlated in NSCLC tumors, yet there is no predicted p53 binding site within the *ODZ4* promoter. Additionally, our TCGA data on p53 is limited, as it is restricted to global DNA and mRNA information. p53 is regulated by post-translational mechanisms and negative regulators, which dictate location and function [67, 68]. Given these data, p53 status and mRNA expression may not be an accurate indicator of p53 activity. Better understanding what transcription factors regulate miR-708-5p expression may help identify the most promising combination therapies that miR-708-5p may add value to. Regardless, we believe it is crucial to advance studies investigating the combinatory tumor suppressive effects of miR-708-5p and chemotherapies *in vivo*.

MiRNAs are attractive therapeutic candidates for treating cancer. They target multiple genes within and across similarly signaling pathways. In theory, this reduces the risk of resistance, as miRNAs more comprehensively suppress oncogenic pathways than a targeted therapy. This is especially relevant in NSCLC, as targeted therapies are effective in only small subsets of patients. While there is enthusiasm for miRNA-focused interventions in cancer, to date their use in the clinic has been limited. There are several hurdles to overcome, specifically concerning efficacy and side effects. The most pressing issue is miRNA delivery, as researchers have struggled to get sufficient amounts of miRNA intratumorally [69]. While ineffective delivery remains a major obstacle, *in vivo* studies using miRNA as single agents have also been underwhelming [70]. Given the current poor therapeutic index for miRNA in solid tumors, their potential as single-agent therapies is limited. Realistically, the likelihood miRNA enter the clinic depends on their synergistic effect with currently approved therapies. Encouragingly, several miRNA have shown promising results when used in combination with chemotherapies [71]. These data bolster the prospect of miR-708-5p as a potentially combinatory treatment. Our data suggest that miR-708-5p can strongly enhance the anti-tumor effects of ERL and PAC. Additionally, while we demonstrate that miR-708-5p combination treatments profoundly regulate proliferation and apoptosis, there are many other hallmarks of cancer left to be studied. It is possible miR-708-5p is involved in immune evasion, as its targets COX-2 and mPGES-1 have notable immunosuppressive functions in cancer. Given these data, we believe there is significant promise in a therapeutic use of miR-708-5p for treating lung cancer. As researchers continue to discover novel targets and uncover new signaling mechanisms, miR-708-5p’s role in lung cancer will be better defined. This will ultimately lead to *in vivo* studies that will better define the complete tumor suppressive function of miR-708-5p in lung cancer.

## MATERIALS AND METHODS

### Bioinformatic and statistical analysis

miR-708-5p predicted targeting sequences were obtained from http://microrna.org/ (website is no longer active). Predicted targets were also analyzed using miRTarBase (http://mirtarbase.mbc.nctu.edu.tw/php/index.php). The Cancer Genome Atlas (TCGA) was mined using the TCGA-assembler 2 R software package [72]. Lung Adenocarcinoma (LUAD) and Lung Squamous Cell Carcinoma (LUSC) RNA-Seq (gene.normalized_RNAseq, gene_RNAseq) and miR-Seq (mir_GA.hg19mirbase20, mir_HiSeq.hg19.mirbase20) were downloaded by TCGA-assembler 2 and analyzed on R using internal lab written software. Clinical data were matched with miR-708-5p expression data and were analyzed using the R packages “survminer” and “survival”. Analyzed data were graphed using “ggplot2”. Significance and confidence intervals were determined using the “survminer” internal *p* value and conf.int functions. These functions compute significance, hazard ratios, and confidence intervals using the log-rank test and 95% upper/lower bands. Inquiries about lab written code can be emailed to carollutzlab@gmail.com. Non-small cell lung cancer (NSCLC) data is a combination of both LUAD and LUSC datasets. The data are expressed as the mean +/– SEM. All non-clinical data are expressed as the mean +/– SD. We used Prism 7 software to perform one-way ANOVA and Student’s *t*-test to determine significant differences. Where indicated, the non-parametric tests were used to determine statistical significance. Inverse correlation studies used the Pearson product-moment correlation coefficient to determine the correlation value, r, and adjusted R^2^. *P*-value was determined by using the correlation coefficient, r, and the sample size. *P*-values less than 0.05 were considered significant.

### Chemotherapeutic treatment

A549 cells were plated in 6-well plates (3 × 10^5^ cells, for protein and RNA isolation) or 60 mm dishes (4 × 10^5^ cells, Ki-67 and Annexin V staining). 24 hours later, cells were expose to serum-containing media plus 10/20 uM erlotinib (low/high) [73], 1/10 nM paclitaxel (low/high) [74, 75], or 1/250 uM dexamethasone (low/high) [76] for 24 hours. Titrations were performed for each therapy based on previous literature (data not shown). We tested the effects on the chemotherapies through 48 hours, but it was determined cells were too stressed to give reliable data beyond 24 hours (data not shown). After 24 hours, media was removed, and RNA/protein were isolated as described in the “RNA Isolation” and “Western Blot Analysis” method subsections.

### Chemotherapeutic resistant cell lines

A549 erlotinib resistant (A549-ER) and paclitaxel resistant (A549-PR) cell lines were created as previously described by Ikeda et al. [64]. Briefly, 5 × 10^5^ A549 cells were plated in 60 mm plates. Once cells were 70% confluent (usually 24 hours later), they were exposed to therapeutic relevant doses of serum-containing DMEM plus erlotinib (25 uM) or paclitaxel (10 nM) for 48 hours. The half-life of erlotinib in culture is ~24 hours, so after 24 hours fresh media plus erlotinib was added. Paclitaxel’s half-life in culture is 48 hours, so we did not replace the media. After 48 hours, cells were washed once with 1X PBS, and fresh serum-containing DMEM was added. Cells were allowed to recover until they were 90% confluent, then were transferred to 150 mm dishes. Once 90% confluent, 5 × 10^5^ A549 cells were seeded in 60 mm dishes while the rest of the cells were taken as frozen stocks. The process was repeated 2 more times for a total of 3 treatments. We confirmed resistance by performing phenotypic assays on our A549-ER and A549-PR cell lines (Supplementary Figures 3–6).

### Mammalian cell culture

A549 cells (ATCC, Manassas, VA, USA) were grown in Dulbecco’s Modified Eagle’s Medium (DMEM, MilliporeSigma) supplemented with 10% FBS, 4 mM L-glutamine, and 1% Penicillin/Streptomycin. All cells were incubated at 37°C in a 5% CO_2_ incubator and sub-cultured using 0.05% Trypsin, 0.53 mM EDTA (Corning, Corning, NY, USA).

### miRNA treatments

A549 cells were seeded in 6-well plates at 3 × 10^5^ cells per well. Synthetic versions of hsa-miR-708-5p and non-targeting miRNAs were purchased from (Horizon Discovery, Waterbeach, UK). Hsa-miR-708-5p mature miRNA sequence: 5′-AAGGAGCUUACAAUCUAGCUGGG-3′, accession #: MIMAT004926. Horizon Discovery’s miRIDIAN microRNA Mimic Negative Control #1 (sequence is not provided) was used as a non-targeting miRNA. This miRNA has a scrambled sequence with no predicted targets in the human transcriptome. Twenty-four hours after seeding, cells were transiently transfected with synthetic miRNAs at 25 nM (unless stated otherwise) using INTERFERin (Polyplus, Berkeley, CA, USA) according to the manufacturer’s protocol. Cells were treated for a total of 48 hours prior to RNA/protein isolation.

### Phenotypic assays

#### Annexin V staining

Apoptosis was measured in A549 cells using the Annexin V Apoptosis Detection I Kit (BS Biosciences). As previously described, A549 cells were plated in 60 mm dishes at 4 × 10^5^ cells per plate. Twenty-four hours later cells were mock or synthetic miRNA (25 nM) treated and returned to grow for 48 hours. Cells were washed with cold 1X PBS then trypsinized (0.25% Trypsin-EDTA, Corning, NY, USA). Cells were centrifuged and resuspended per manufacturer’s protocol. Following resuspension, appropriate amounts of phycoerythrin (PE) labeled Annexin V and DAPI were added to 2 × 10^5^ cells and incubated for 15 minutes in the dark. Samples also included an unstained negative control and boiled positive control. Flow Cytometry was performed on the BD FACSCelesta machine (BD Biosciences), recording 20,000 events per sample. Data were analyzed using FlowJo software (BD Biosciences). Analysis revealed alive, early apoptotic, and late apoptotic/necrotic populations as previously shown by Wallberg et al. [77].

#### Ki-67 staining

Proliferation was measured in A549 cells using the FITC Mouse Anti-Ki-67 Kit (BS Biosciences, San Jose, CA, USA). A549 cells were plated in 60 mm dishes at 4 × 10^5^ cells per plate. Twenty-four hours later cells were mock or synthetic miRNA (25 nM) treated and returned to grow for 48 hours. Cells were washed with cold 1X PBS then trypsinized (0.25% Trypsin-EDTA, Corning, NY, USA). Cells were then fixed per the manufacturer’s protocol and put in –20°C for a minium of 2 hours. Following the manufacturer’s guidelines, Ki-67 and propidium iodide (PI) were added to 1 × 10^6^ cells and incubated. Samples also include an IgG isotype control that stains negative for Ki-67. Flow Cytometry was performed on the BD FACSCelesta machine (BD Biosciences), recording 30,000 events per sample. Data were analyzed using FlowJo software (BD Biosciences). The alive population was selected from each sample (forward versus side scatter). Further analysis revealed Ki-67 +/– populations, as well as cell cycle stage as previously done by Kim & Sederstrom [78].

### Quantitative real-time PCR (qRT-PCR)

Complementary DNA (cDNA) was synthesized by reverse transcription of RNA using the miScript II RT Kit (Qiagen, Venlo, Netherlands). miRNA specific cDNA was created using HiSpec buffer, while mRNA specific cDNA was created using HiFlex buffer. qRT-PCR was performed using a Bio-Rad CFX96 Real-Time C1000 Touch Thermal Cycler. MiRNA cycling conditions were as follows: (1) 95°C for 15 min; (2) 40 cycles of 94°C for 15 sec, 55°C for 30 sec, 70°C for 30 sec (collection step). mRNA cycling conditions were similar, except for adjusted annealing temperatures on a primer-by-primer basis. miR-708-5p, U6 snRNA, miR-15a, COX-2, and mPGES-1 primers were purchased from Qiagen, while ODZ4 and GAPDH primers were purchased from Origene. Amplification was performed using the miScript SYBR Green PCR Kit (Qiagen). No template and no reverse transcriptase controls, as well as melt curve analysis, were implemented to ensure samples/primers were not contaminated. Quantitative Comparative C_T_ (ΔΔC_T_) analysis was used to analyze gene expression changes relative to U6 snRNA/miR-15a (miRNA) or GAPDH (mRNA). qRT-PCR data represent the average of ≥ 3 biological replicates. Each sample was measured with *n* ≥ 2 technical replicates per target gene per independent experiment.

### RNA isolation

Total RNA was isolated from cells using TRIzol (Invitrogen, Carlsbad, CA, USA) following the manufacturer’s protocol. Samples were further purified with the Direct-zol RNA Miniprep Kit (Zymo Research). RNA was quantified using the Simpli-Nano Spectrophotometer (GE, Boston, MA, USA).

### Western blot analysis

Media was removed and treated cells were lysed in RIPA buffer (50 mM Tris at pH 8.0, 150 mM NaCl, 1% Nonidet P-40, 0.5% sodium deoxycholate, 0.1% SDS, 0.1% protease inhibitor). The cells were scraped off wells, collected, then centrifuged at 14000 × g for 15 min at 4°C. Protein concentration was determined using the DC Protein Assay (Bio-Rad, Hercules, CA, USA). 25 ug of protein were loaded onto 10% SDS-PAGE gels, separated by electrophoresis, and transferred onto PVDF membrane (VWR) for 2 hours at 4°C. Blots were blocked with 5% non-fat milk + PBSt (5% non-fat dry milk, 1× PBS, 0.1% Tween-20 [MilliporeSigma]) for 1 hour at room temperature (RT). Primary antibody incubations against human COX-2 (160112, Cayman Chemical, Ann Arbor, MI, USA), CHOP (L63F7, Cell Signaling), mPGES-1 (ab180589, Abcam), Survivin (ab76424, Abcam), p53 (05-224, MilliporeSigma), and GAPDH (HRP-60004, Proteintech, Rosemont, IL, USA) were performed overnight at 4°C per manufacturer’s recommended dilutions. Blots were washed with PBSt 3× for 5 minutes each, then exposed to secondary HRP conjugated secondary antibodies (Goat anti-Mouse H+L [31430, ThermoFisher, Waltham, MA, USA], Goat anti-Rabbit H+L [31460, ThermoFisher]) for 1 hour at RT. Blots were developed using Clarity Western ECL Substrate (Bio-Rad) on the ChemiDoc MP Imaging system (Bio-Rad). Western blot images are representative of ≥ 3 biological replciates.

### Note

microrna.org is no longer an active site, but it was in the past.

## SUPPLEMENTARY MATERIALS



## Abbreviations

A549-ER: A549 erlotinib resistant
A549-PR: A549 paclitaxel resistant
AA: arachidonic acid
ALDH1A2: aldehyde dehydrogenase 1 family member A2
cDNA: complementary DNA
CHOP: CCAAT enhancer-binding protein homologous protein
COX-1: cyclooxygenase-1
COX-2: cyclooxygenase-2
CSC: cancer stem cell
DEX: dexamethasone
ERL: erlotinib
LUAD: lung adenocarcinoma
LUSC: squamous cell carcinoma of the lung
MAPK: mitogen-activated protein kinase
miR-708-5p: miR-708
miRNA: microRNA
mPGES-1: microsomal prostaglandin E synthase 1
MRP4: multidrug resistance-associated protein 4
NF-kB: nuclear factor kappa-light-chain enhancer of activated B cells
NSCLC: non-small cell lung cancer
PAC: paclitaxel
PE: phycoerythrin
PGE_2_: prostaglandin E2
PGES: prostaglandin E synthase
PGH_2_: prostaglandin H2
PI: propidium iodide
PI3K: phosphoinositide 3-kinase
PS: phosphatidylserine
qRT-PCR: quantitative real-time polymerase chain reaction
RT: room temperature
UTR: untranslated region
