# Supplementary material for: miR-708-5p enhances erlotinib/paclitaxel efficacy and overcomes chemoresistance in lung cancer cells
Source: Oncotarget. 2020 Dec 22;11(51):4699–721. doi: 10.18632/oncotarget.27840 (PMC7771713; doi:10.18632/oncotarget.27840)
Supplement: Supplementary file 1 [file oncotarget-11-4699-s001.pdf]

miR-708-5p enhances erlotinib/paclitaxel efficacy and overcomes chemoresistance in lung cancer cells

SUPPLEMENTARY MATERIALS

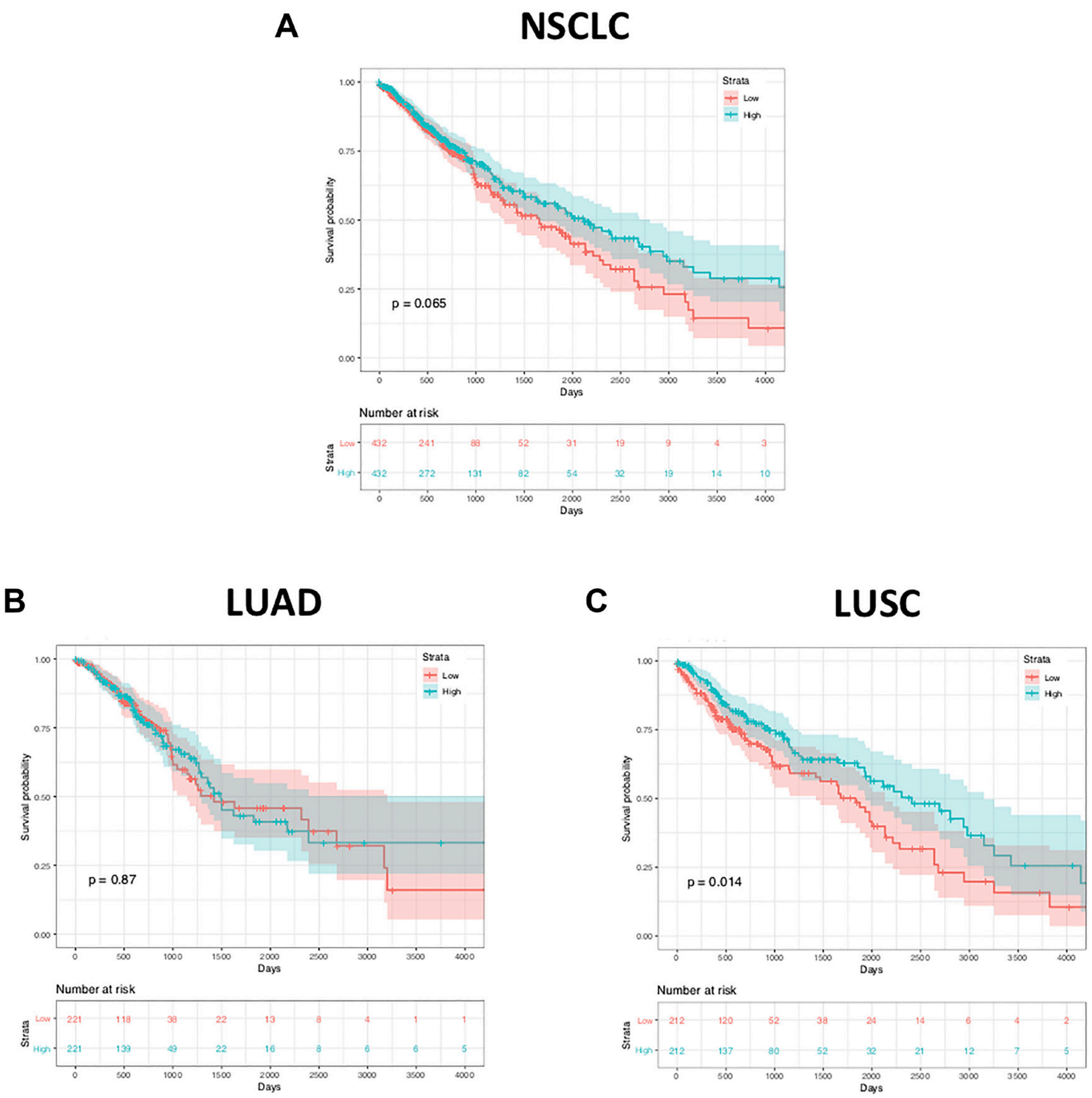

Supplementary Figure 1: CHOP mRNA expression is associated with increased survival rates in LUSC patients. Kaplan-Meier plots from TCGA data measuring the effects of high (blue) or low (red) CHOP mRNA expression in (**A**) NSCLC (p = 0.065, n = 864), (**B**) LUAD (p = .87, n = 442), and (**C**) LUSC (p = .014, n = 424), on patient survival rates. The bottom of each graph indicates the number of patients at risk for each time point.

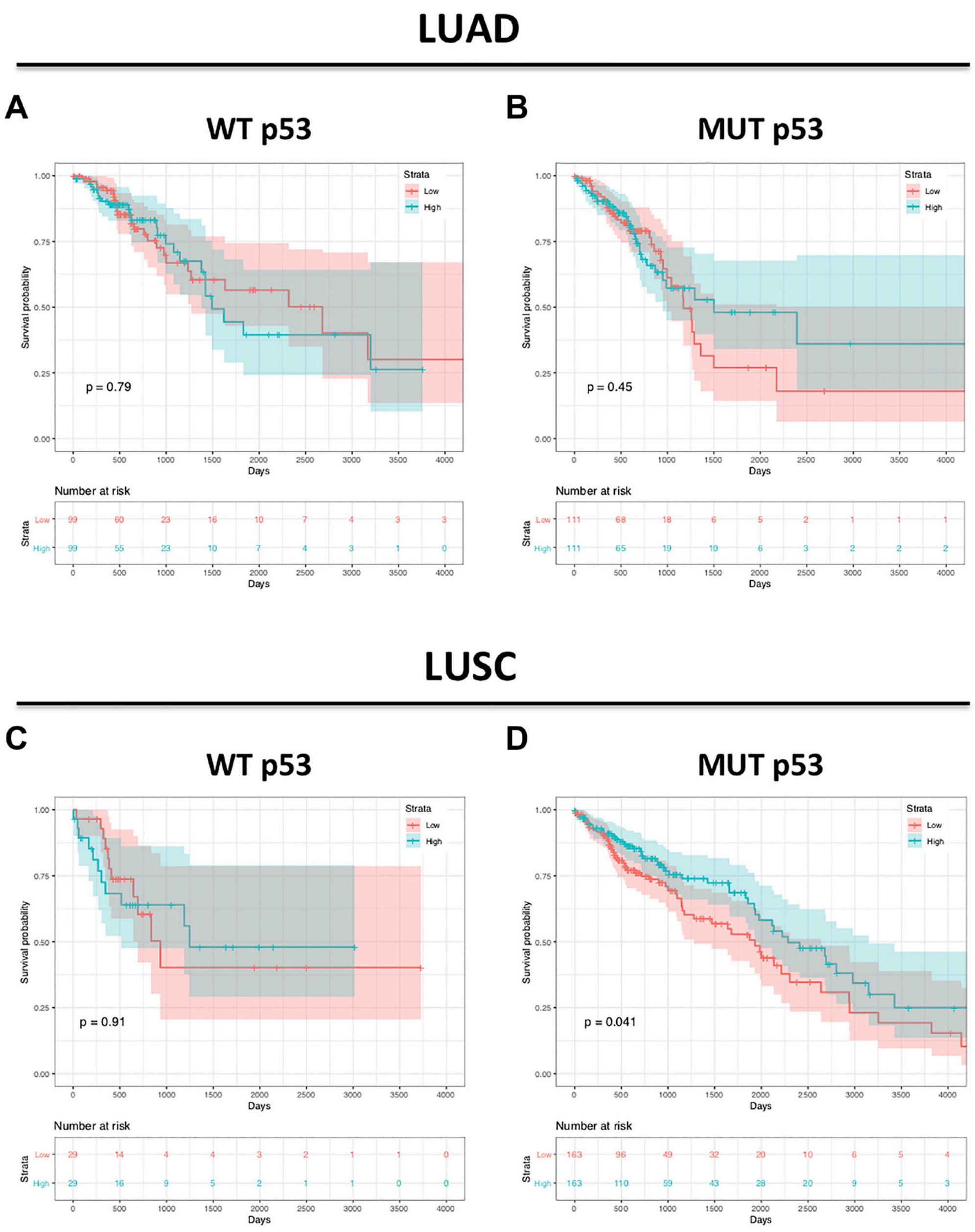

Supplementary Figure 2: miR-708-5p increases survival rates in p53 mutant LUSC tumors. Kaplan–Meier plots from TCGA data measuring the effects of high (blue) or low (red) mature miR-708-5p expression in LUAD (**A**) WT p53 ($p = .79$, $n = 240$) and (**B**) MUT (oncogenic + likely oncogenic mutants [$p = .45$, $n = 263$]) patient survival rates. We also examined the effects of high (blue) or low (red) mature miR-708-5p expression in LUSC (**C**) WT p53 ($p = .91$, $n = 70$) and (**D**) MUT (oncogenic + likely oncogenic mutants [$p = .041$, $n = 389$]) patient survival rates. The bottom of each graph indicates the number of patients at risk for each time point.

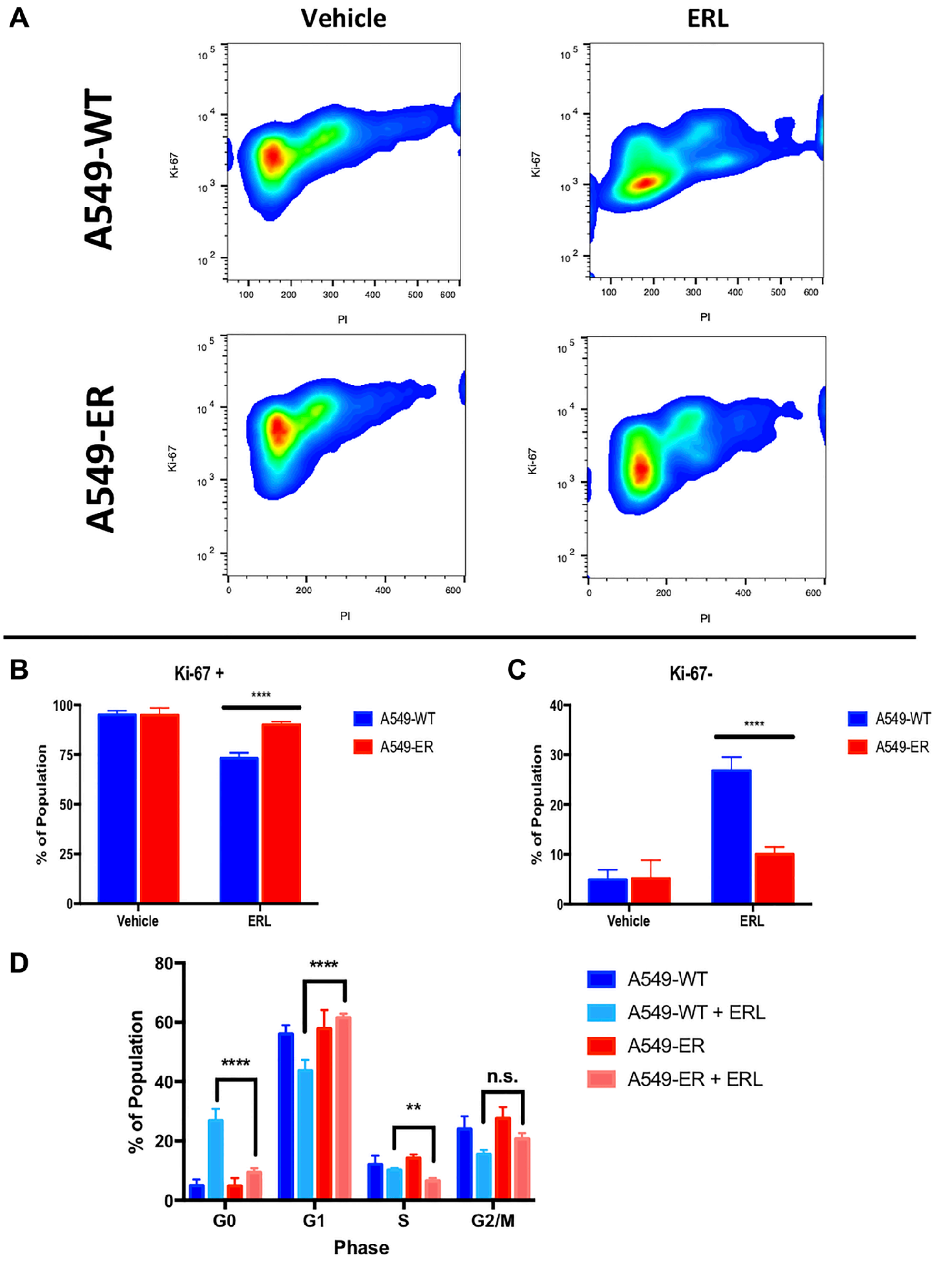

Supplementary Figure 3: A549-ER cell proliferation is nonresponsive to ERL treatment. (**A**) Representative smoothed graph of flow cytometry data showing cell cycle stage based on Ki-67 (y-axis) and PI staining (x-axis) in A549-WT cells treated with vehicle (top left) or 20 uM ERL (top right), as well as A549-ER cells treated with vehicle (bottom left) or 20 uM ERL (bottom right) for 48 hours. (**B** and **C**) Quantification of (B) Ki-67 positive (< 10^3) and (C) negative (>10^3) populations in A549-WT (blue) and A549-ER (red) cells. (**D**) Cell cycle quantification of samples treated with vehicle (A549-WT [blue], A549-ER [red]), or 20 uM ERL (A549-WT [light blue], A549-ER [light red]). $^{**}p < .01$, $^{****}p < .0001$, n.s. = not significant, $n \geq 3$.

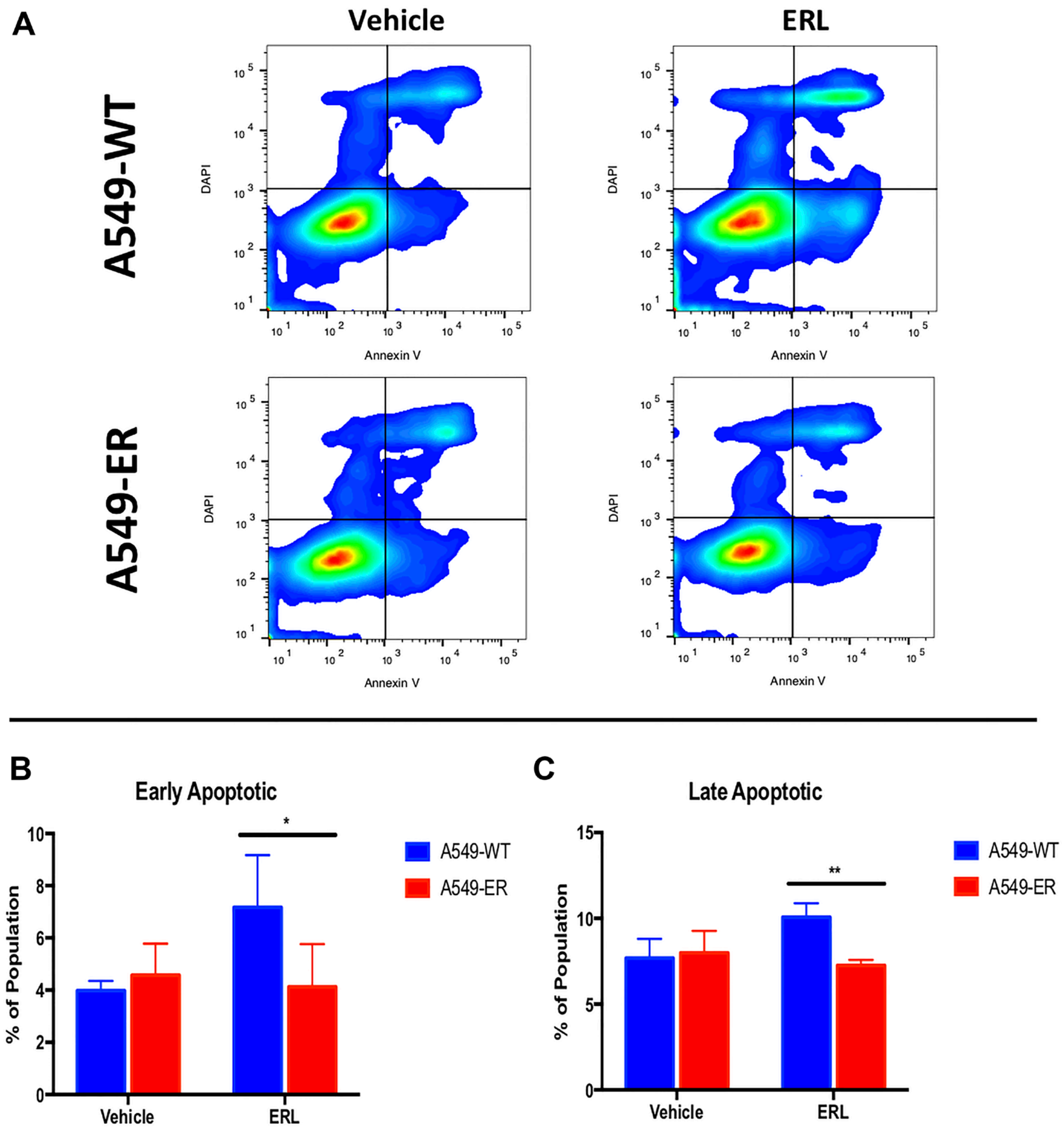

Supplementary Figure 4: ERL does not induce apoptosis in A549-ER cells. (**A**) Representative smoothed graphs of flow cytometry data from Annexin V and PI stained A549-WT cells treated with vehicle (top left) or 20 uM ERL (top right), as well as A549-ER cells treated with vehicle (bottom left) or 20 uM ERL (bottom right) for 48 hours. (**B**) Quantification of the early apoptotic (Annexin V+, DAPI-) populations in A549-WT (red) and A549-ER (blue) cells from (A). (**C**) Quantification of the late apoptotic (Annexin V+, DAPI+) populations in A549-WT (red) and A549-ER (blue) cells from (A). $^{*}p < .05$, $^{**}p < .01$, $n \geq 3$.

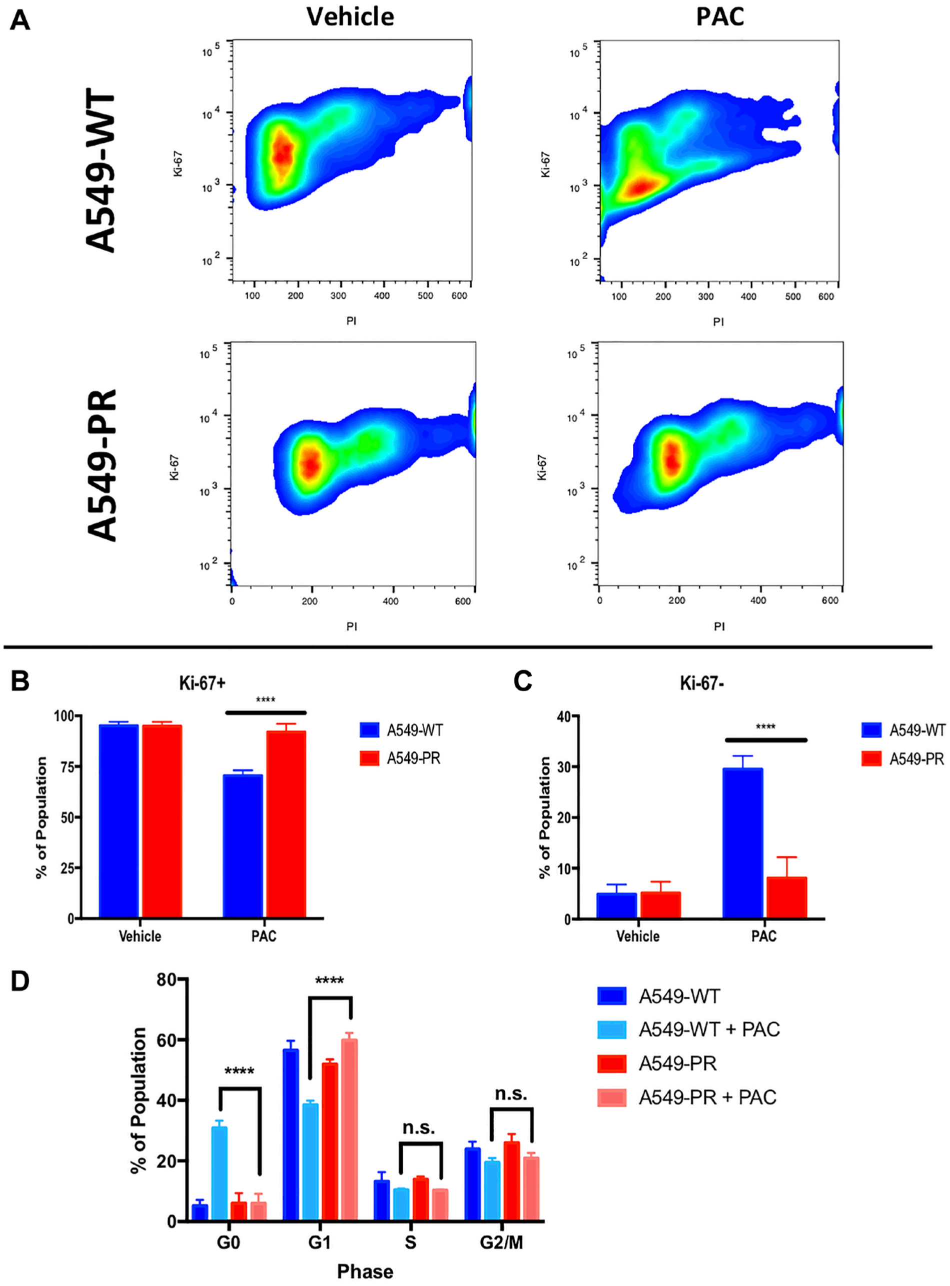

Supplementary Figure 5: PAC does not alter A549-PR cells proliferation rates. (**A**) Representative smoothed graph of flow cytometry data showing cell cycle stage based on Ki-67 (y-axis) and PI staining (x-axis) in A549-WT cells treated with vehicle (top left) or 10 nM PAC (top right), as well as A549-PR cells treated with vehicle (bottom left) or 10 nM PAC (bottom right) for 48 hours. (**B** and **C**) Quantification of (B) Ki-67 positive (< 10^3) and (C) negative (> 10^3) populations in A549-WT (blue) and A549-PR (red) cells. (**D**) Cell cycle quantification of samples treated with vehicle (A549-WT [blue], A549-PR [red]), or 10 nM PAC (A549-WT [light blue], A549-PR [light red]). $^{****}p < .0001$, n.s. = not significant, $n \geq 3$.

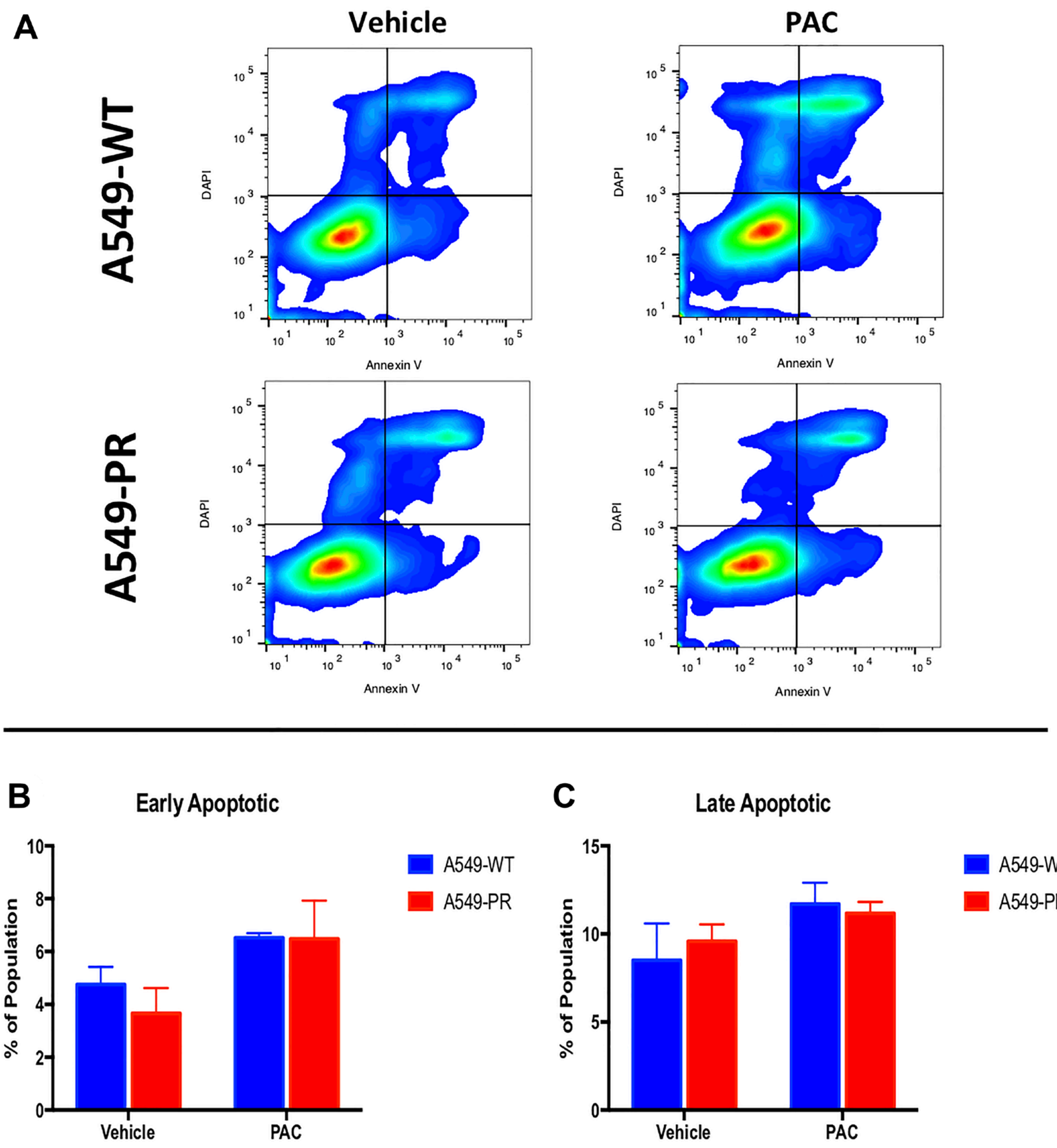

Supplementary Figure 6: PAC does not induce apoptosis in A549-PR cells. (**A**) Representative smoothed graphs of flow cytometry data from Annexin V and PI stained A549-WT cells treated with vehicle (top left) or 10 nM PAC (top right), as well as A549-PR cells treated with vehicle (bottom left) or 10 nM PAC (bottom right) for 48 hours. (**B**) Quantification of the early apoptotic (Annexin V+, DAPI-) populations in A549-WT (red) and A549-PR (blue) cells from (A). (**C**) Quantification of the late apoptotic (Annexin V+, DAPI+) populations in A549-WT (red) and A549-PR (blue) cells from (A). $n \geq 3$.
